# A miniature piezoelectric-thermomagnetic generator for low-grade waste heat recovery

**DOI:** 10.1038/s41598-026-59705-2

**Published:** 2026-07-31

**Authors:** Maxim Wischnewski, Joel Joseph, Makoto Ohtsuka, Hiroyuki Miki, Manfred Kohl

**Affiliations:** 1 Institute of Microstructure Technology, Karlsruhe Institute of Technology (KIT), Karlsruhe, Germany; 2 Institute of Multidisciplinary Research for Advanced Materials, Tohoku University, Sendai, Japan; 3 Faculty of Science and Engineering, Ishinomaki Senshu University, Miyagi, Japan

**Keywords:** Power generation, Waste heat recovery, Thermomagnetic energy harvesting, Magnetic shape memory film, Ni-Mn-Ga, Piezoelectric layer, PZT, Energy science and technology, Engineering, Materials science, Nanoscience and technology, Physics

## Abstract

This paper presents the experimental and model-based investigation of a compact miniature-size generator for conversion of low-grade waste heat at temperatures near 100 °C to electricity leveraging the combined transducer effects of a thermomagnetic (TM) and a piezoelectric (P) layer. TM energy conversion is achieved via the recently developed effect of resonant self-actuation of a cantilever using the large temperature-induced change of magnetization at the ferromagnetic transition of a Ni-Mn-Ga film. The resulting kinetic energy is converted into electrical energy via the piezoelectric effect of a thin lead-zirconium-titanate (PZT) layer, which enables rectification of the harvested energy using standard electrical circuitry. The performance of the generator is characterized with respect to the effects of tip mass and length of the piezoelectric layer as well as heat transfer and damping. A lumped element model (LEM) of the coupled system shows that heat intake and heat dissipation have to be balanced to achieve resonant self-actuation, while damping limits the oscillation frequency of the cantilever. A single P-TM generator generates an average power output of 0.8 µW at 105 °C. After rectification, this corresponds to 1.25 µW/cm² of DC power at a voltage of 2 V DC.

**Supplementary Information:**

The online version contains supplementary material available at 10.1038/s41598-026-59705-2.

## Introduction

Waste heat is naturally available as geothermal, volcanic or solar heat^4^ and generated due to inefficiencies in appliances and industrial processes^1–3^. Estimates suggest that over 60% of the world’s primary energy supply ends up dissipated into the environment, primarily as heat^1^. In particular, heat sources at temperatures of 100 °C and below are abundant, offering a nearly untapped resource for energy recovery, which is often neglected due to low conversion efficiencies^1^. In this realm, miniaturized thermal energy harvesters open up novel opportunities to convert such low-grade waste heat into electricity. This may enable new routes for supplying autonomous sensor systems for the Internet of Things (IoT), portable electronic devices or wearables with electricity^1,4,5^. By continuously recharging, they can extend lifetime and, thus, reduce reliance on batteries enabling maintenance-free operation in remote or hard-to-reach environments^4,6^.

Currently, thermoelectrics is the most advanced technology. Thermoelectric generators are solid-state devices for converting heat into electricity via the Seebeck effect^1,4–7^. However, at low temperature differences, the figure of merit of thermoelectric materials becomes very low^6^. At the same time, maintaining a large temperature gradient across the thermoelectric generator remains challenging^1^, requiring large heat sinks or active cooling. An alternative are TM generators making use of the large, reversible change of magnetization in TM materials at their Curie temperature ($\:{T}_{C}$). This approach has gained increasing attention recently due to the availability of materials with enhanced TM properties^8^. TM materials exhibit a steep change in magnetization $\:\varDelta\:M/\varDelta\:T$, which allows to drive a highly efficient thermomagnetic cycle. The best TM materials can reach an efficiency up to 2%, which is about 60% of the upper limit of Carnot efficiency of 3.3% for a temperaure difference $\:\varDelta\:T$ of 10 K and temperature at the hot side of 300 K^8^. Different approaches of TM generation have been presented, which differ by the method of heat transfer and conversion of thermal energy into electricity^4,9–14^. TM generators either convert the thermal energy directly using the magnetization change of the material or indirectly using a periodic mechanical motion generated from the magnetization change of the material^5,15^.

Recent macro-scale devices make use of a heat transfer fluid to enable rapid temperature changes of the TM material placed in a magnetic circuit. The resulting changes of magnetic flux are then used to generate a current in a pick-up coil according to Faraday’s law^1,5^. A TM generator featuring a magnetic circuit with pretzel-like topology is presented in^10^. This device utilizes two stacks of La–Fe–Co–Si plates functioning as thermally activated magnetic flux switches. An alternative TM generator design is presented in^1^ using a 3D printed sample cabin placed in the center of a hollow cylindrical Halbach permanent magnet.

Miniature-scale devices make use of TM films or TM foils with large surface-to-volume ratio allowing for rapid heat transfer through solid-solid contact to heat sink and source^12,16–18^. Thus, fluid pumps and circuitry are avoided allowing for compact system designs. High performance TM generators at miniature scale have been developed based on the concept of resonant self-actuation^12^. This concept relies on the periodic magnetic attraction of a TM film placed at the front of a cantilever caused by the field gradient of a permanent magnet, which at the same time acts as the heat source. The resulting resonant oscillation of the cantilever in the order of 100 Hz effectively converts thermal energy into kinetic energy, which is subsequently converted into electricity using a pick-up coil. By up-scaling the TM film thickness and geometry optimization, a large power per footprint of 50 µW/cm² has been demonstrated^14^. Resonant self-actuation occurs at small temperature differences of a few Kelvins enabling completely autonomous operation and simplified heat management^1,5^. In the meantime, resonant self-actuation has been demonstrated for different TM film materials including Ni-Mn-Ga, Gd and LaFeSi^14,19,20^.

Converting kinetic energy into electricity by induction typically generates low AC voltages and, thus, conversion to usable DC power is challenging^21^. This challenge can be avoided by piezoelectric transduction, which inherently produces high voltages facilitating voltage rectification. The mechanical-to-electrical conversion efficiency of cantilevered piezoelectric harvesters at resonance approach 44% in the limit of high electromechanical coupling^22^. Cantilever based piezoelectric devices have been widely studied in kinetic energy harvesting systems^21,23–27^. Here, we combine the high efficiency of TM film-induced resonant self-actuation with piezoelectric conversion of the resulting kinetic energy into electrical energy. This enables rectification and energy storage by standard electrical circuitry, opening up a novel route to generate usable DC power from low-grade waste heat. A lumped-element model (LEM) is developed to investigate the effects of heat transfer and damping. Several demonstrators of the piezoelectric-thermomagnetic generator (P-TM generator) with increasing length of the P layer are fabricated and characterized with respect to their coupled thermal, mechanical and electrical performance.

## Results

### Material properties

**Fig. 1:**
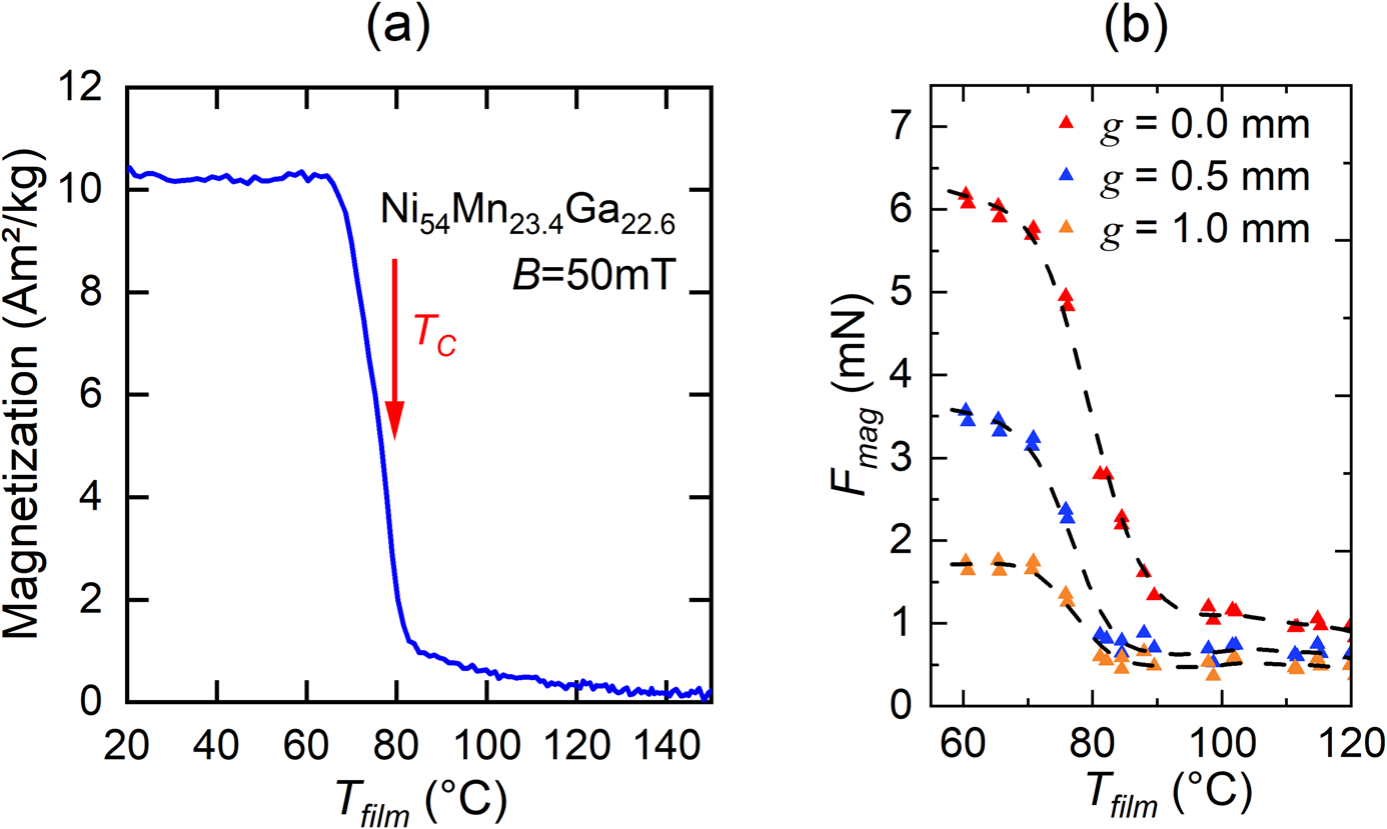
Magnetic properties of the Ni-Mn-Ga films. (**a**) Magnetization versus temperature measurement of a Ni-Mn-Ga film at a constant magnetic field of 50 mT, showing a steep magnetization change at 80 °C. (**b**) Experimentally determined magnetic attraction force between a 2 × 2 × 0.01 mm³ Ni-Mn-Ga film and a permanent magnet of size 3 × 3 × 8 mm³ and a remanent magnetic field of 1.08 T, as a function of film temperature at different gap size between film and magnet.

The TM material utilized in this investigation is a Heusler alloy Ni-Mn-Ga film. Heusler alloys belong to the class of intermetallic compounds, which are characterized by their ordered L2_1_ type crystal structure^28^. Ni-Mn-Ga is well known for exhibiting both ferromagnetic as well as ferroelastic properties showing strong coupling of crystallographic structure and magnetic ordering^29^. Ni-Mn-Ga films of 10 μm thickness are deposited on a polyvinyl alcohol substrate by RF magnetron sputtering at room temperature using a sputtering power of 200 W^30^. As-deposited films are amorphous and, thus, require a heat treatment, which is performed at 800 °C for 36 ks. The chemical composition of the films is determined by the inductive plasma method to be Ni_54_Mn_23.4_Ga_22.6_. Here, the ferromagnetic transition in the Ni-Mn-Ga film is of special interest. Fig. 1(a) shows the temperature-dependent magnetization at low magnetic field of 50 mT. At the Curie temperature of about 80 °C, the magnetization exhibits a sharp change ΔM in a narrow temperature window ΔT with a maximum slope *ΔM*/*ΔT* of 2.3 Am^2^(kgK)^−1^. This will be exploited for TM actuation in the following by modulating the magnetic attraction force $\:{F}_{mag}$ between the film and a permanent magnet shown in Fig. 1(b) through a change of film temperature $\:{T}_{film}$.

The piezoelectric material used for energy conversion is a polycrystalline lead zirconate titanate (PZT) plate of 100 μm thickness that has been thinned down to 30 μm by mechanical polishing. PZT materials have a perovskite crystal structure and exhibit large piezoelectric coefficients in compositions near the morphotropic phase boundary, which is characterized by the coexistence of rhombohedral and tetragonal phases enhancing the piezoelectric response^31,32^. The high Curie temperature of PZT materials prevent temperature induced depolarization^32^, making them suitable for applications at elevated temperatures. Here, a commercially available bulk PZT plate is used with transverse piezoelectric coefficient $\:{d}_{31}$ of -315∙10^− 12^ C/N, compliance $\:{s}_{11}$ of 14.2∙10^− 12^ m²/N and a relative dielectric constant $\:{\epsilon\:}_{33}$ of 4500, resulting in a transverse electro-mechanical coupling coefficient $\:{k}_{31}$ of 0.42.

### Device layout and setup

**Fig. 2:**
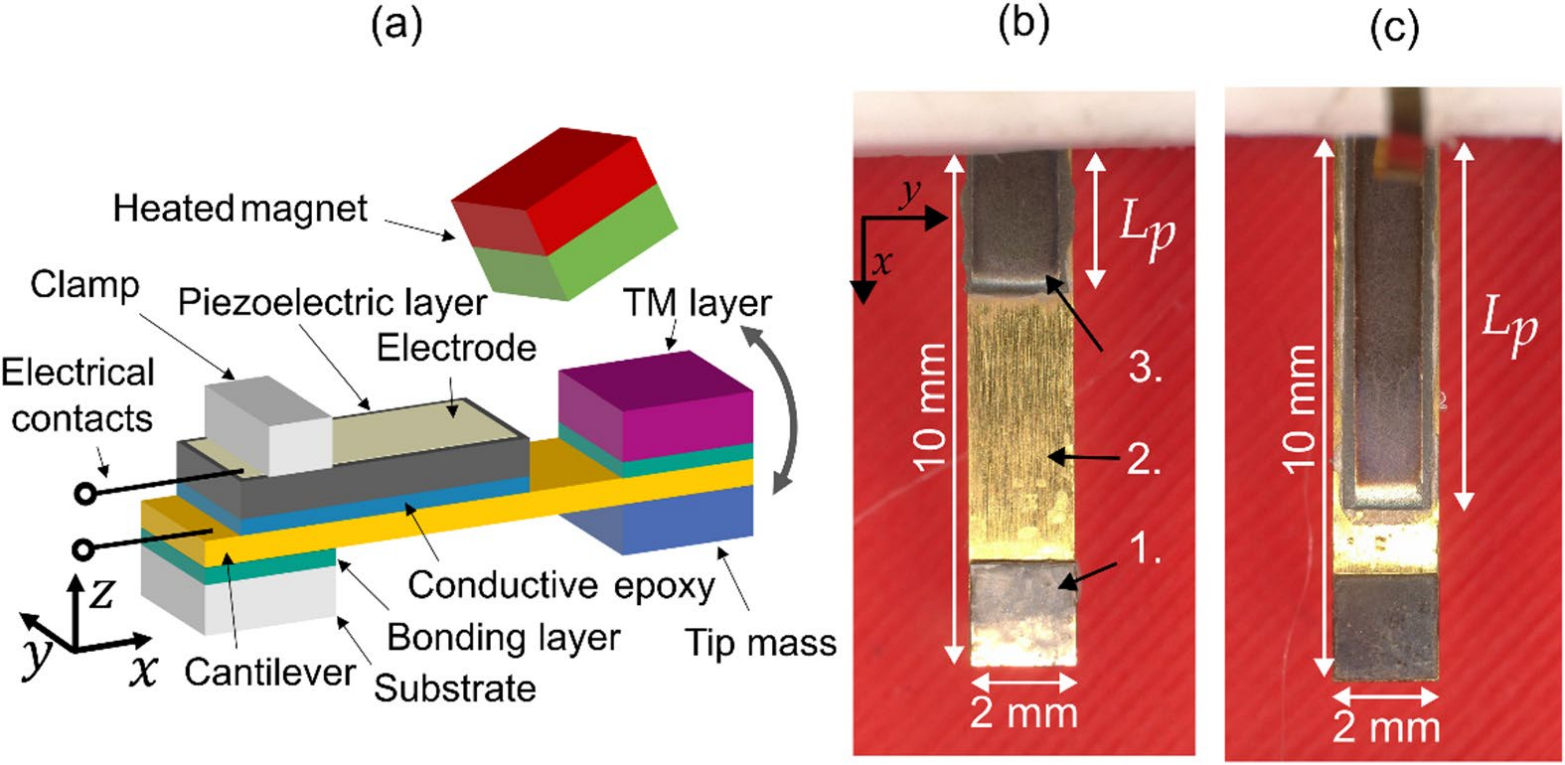
Layout of the P-TM generator. (**a**) Schematic drawing; (**b**) and (**c**) photos of two demonstrator devices (top view) with different lengths of the piezoelectric layer of $\:{L}_{p}$ 3 and 7 mm, respectively. Legend: (1) TM layer; (2) cantilever, (3) piezoelectric layer with top electrode.

Figure. 2(a) depicts a schematic of the multilayer P-TM generator, which comprises two functional layers for energy conversion: a soft-magnetic layer of two stacked Ni-Mn-Ga films bonded to the front of a cantilever after heat treatment and a piezoelectric layer of PZT bonded to the onset of the cantilever. The cantilever is made of metal with sufficiently high thermal conductivity, such as CuZn. Above the TM layer, a heated permanent magnet of SmCo provides for a large gradient of magnetic field and, at the same time, acts as the heat source for thermal energy harvesting. Further details on the experimental setup are presented in the Methods section. The materials and geometrical parameters are designed to enable resonant self-actuation of the cantilever. When the temperature of the TM layer is below $\:{T}_{C}$, the magnetization of the TM layer and the magnetic field gradient result in a magnetic attraction force bending the cantilever towards the heated magnet. At contact, the TM layer’s temperature increases resulting in a reduction in magnetization and consequently in magnetic attraction force. Once the cantilever’s elastic restoring forces become dominant, the TM layer loses contact to the heat source and swings back towards its initial position. Thereby, it cools down by heat conduction via the metal layer and heat convection. Once the magnetization of the TM layer is sufficiently restored, the actuation cycle restarts resulting in self-actuation of the cantilever as long as thermal energy is available. When the heat intake and dissipation within a cycle are balanced with the mechanical cycle, resonant self-actuation is achieved, in which case a continuous oscillation occurs with constant amplitude over time. Due to the short duration of each cycle, the heating time at contact is in the order of milliseconds, while the temperature change can be as low as a few Kelvin^14^.

The piezoelectric layer is designed to convert the kinetic energy of the oscillating cantilever into electrical energy making use of the bending-induced mechanical stress. A fabrication strategy is adopted that includes mechanical thinning of the PZT plate to a final thickness of 30 μm. The PZT plate is bonded onto the cantilever using a conductive epoxy layer. To maximize stress, the PZT layer is clamped to the supporting substrate to increase rigidity at the fixed end. The benefits of integrating bulk materials through bonding techniques with subsequent thinning by mechanical grinding have been demonstrated, e.g., in^23,26,33^. The design includes an additional tip mass to tune the cantilevers’ natural frequency, matching the mechanical and thermal cycle times required for resonant self-actuation. Table 1 in the Methods section summarizes the material parameters. The geometrical parameters of the devices under test are given in Table S1 in the Supplementary Information. Several P-TM generators with varying length of the piezoelectric layer ($\:{L}_{p}$), between 3 and 7 mm, are characterized with respect to thermomagnetic actuation amplitudes and frequencies, as well the electrical power output. Measurement details are given in the Methods section.

### Lumped-element model

A lumped element model (LEM) approach is used to describe the thermo-magneto-mechanical and electromechanical coupling effects in the P-TM generator. Thereby, the details on spatial temperature gradients and non-uniform stress distributions are subsumed in effective adjustment parameters allowing for a computationally efficient approach to describe the dynamic performance of the P-TM generator and its parameter dependencies at the system level. The procedure is detailed in the Methods section.

### Coupled thermo-magneto-mechanical performance

The coupling of the different sub-models allows to match simulation and experimental results and, thus, to investigate the conditions for resonant self-actuation as well as identifying the optimal performance parameters for maximum power during resonant self-actuation. This is demonstrated in the following for the time-resolved mechanical performance of a P-TM generator having a piezoelectric layer of length $\:{L}_{p}$ = 7 mm for a constant source temperature $\:{T}_{source}$ of 100 °C.

**Fig. 3:**
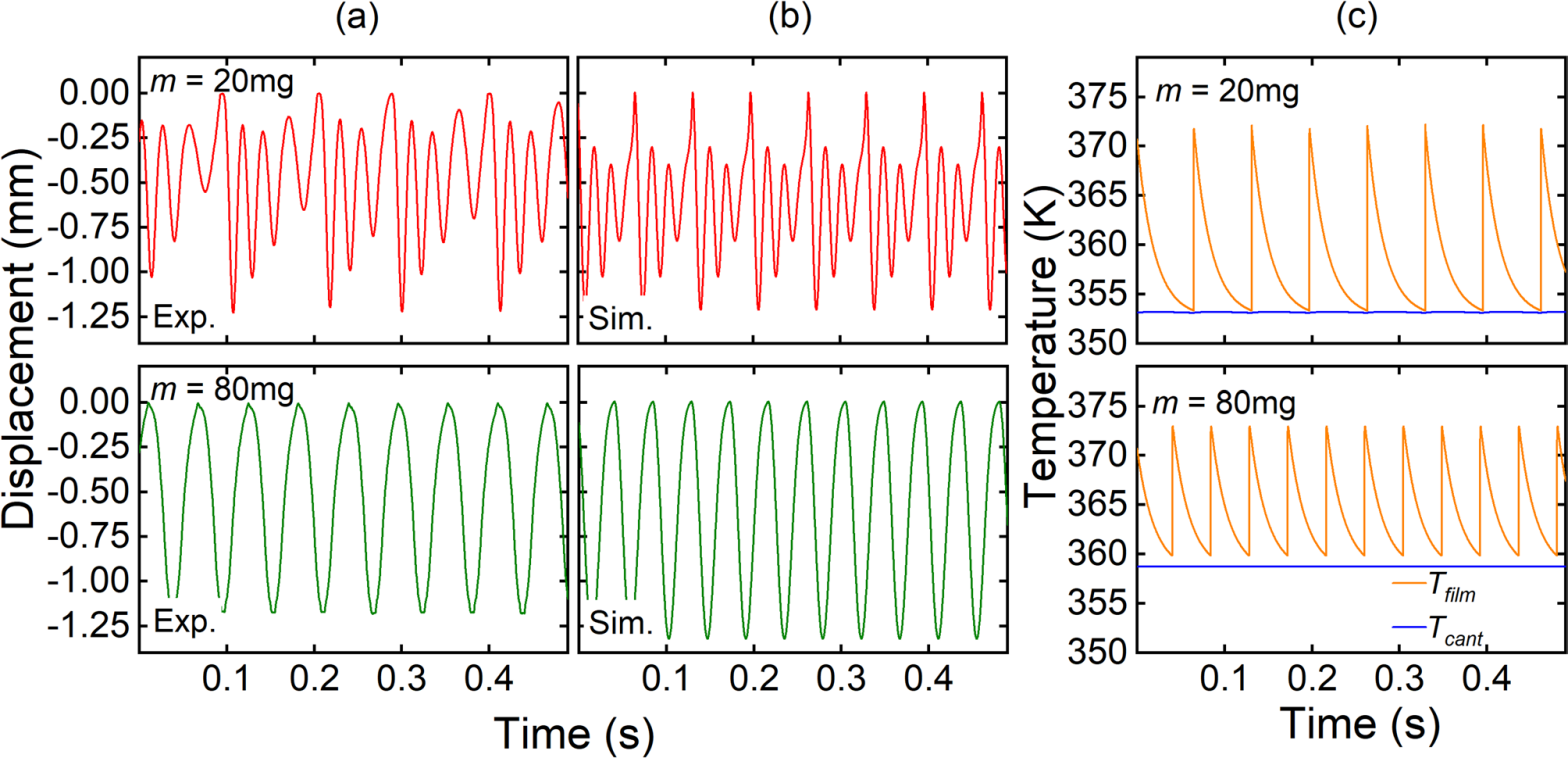
Time-resolved displacement characteristics of the cantilever tip and corresponding simulated course of average temperatures of the TM layer for a P-TM generator with piezoelectric layer of length $\:{L}_{p}$ = 7 mm at a source temperature $\:{T}_{source}$ of 100 °C. Experiment characteristics (**a**) and LEM simulations (**b**) are shown for a tip mass of 20 and 80 mg. The simulations qualitatively capture the oscillation behavior in the two scenarios. Resonant self-actuation is observed for a tip mass of 80 mg indicating that the cooling time needed to regain sufficient magnetic attraction matches the time duration of mechanical oscillation. The LEM-simulations of TM layer temperature (**c**) reflect the thermal duty cycles for the tip mass of 20 and 80 mg. Legend: $\:{T}_{film}$ – temperature of TM layer, $\:{T}_{cant}$ – temperature of cantilever.

Figure 3(a) shows experimentally determined displacement characteristics of the cantilever tip for a tip mass of 20 and 80 mg. For the tip mass of 20 mg, the oscillation amplitude decays roughly by a factor of 2 within three oscillation cycles starting from an initial amplitude of about 1.25 mm. After about 100 ms, the cantilever tip returns to the surface of the magnet indicating that the magnetic attraction becomes sufficiently strong again to pull the TM layer back and to repeat this sequence.

For the tip mass of 80 mg, however, resonant self-actuation occurs, which is reflected by the continuous oscillation of about 17 Hz with large constant amplitude. This performance is well reproduced by the LEM simulations shown in Fig. 3(b) even though the simulated oscillation stroke and frequency of resonant oscillation are larger to some extent. In the case of resonant self-actuation, the frequency of the thermal cycle matches the mechanical oscillation frequency, i.e., the heating time at contact to the heat source (permanent magnet) allows for a sufficient temperature increase for the cantilever to retract and the cooling time allows for sufficient temperature decrease to regain sufficient magnetic attraction in order to sustain continuous oscillation.

The corresponding simulated temperature changes are displayed in Fig. 3(c). For the tip mass of 20 mg, a considerably longer cooling time is needed to recover sufficient magnetic attraction for the contact with the magnet compared to the case of 80 mg. This is due to the larger oscillation frequency and corresponding shorter mechanical oscillation cycle, during which the temperature does not decrease sufficiently. Consequently, the needed cooling time extends over three oscillations, whereby the average temperature of the TM layer reaches a minimum of approximately 80 °C. In the case of resonant self-actuation, the temperature change during one oscillation cycle is about 13 K corresponding to a minimal temperature of the TM layer of approximately 86 °C. Under stationary conditions, the corresponding temperature of the cantilever remains almost constant at about 85 °C. In summary, we can infer that increasing the tip mass reduces the oscillation frequency and, thus, provides more time for the TM layer to cool and for the magnetic force to recover. In addition, the influence of damping has to be considered, as will be further discussed in the following.

**Fig. 4:**
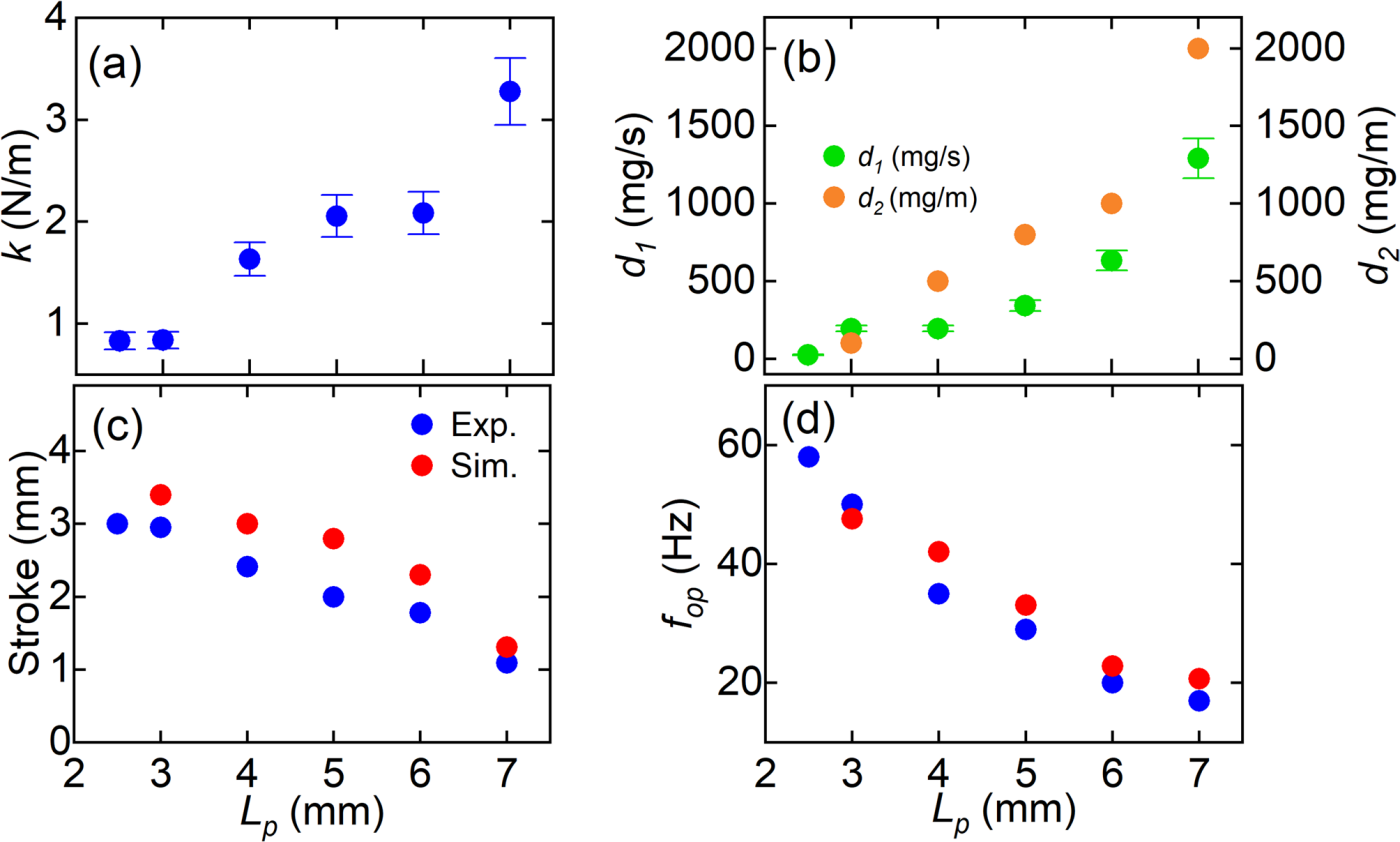
Mechanical performance metrics of the P‑TM generators as a function of the piezoelectric‑layer length $\:{L}_{p}$. (**a**,** b**) Effective stiffness *k* and viscous ($\:{d}_{1}$) / quadratic ($\:{d}_{2}$) damping coefficients of the cantilever determined from step-response experiments (see Fig. S1 in the Supplementary Information) versus the length of piezoelectric layer $\:{L}_{p}$. (**c**) Experimental and simulated stroke of cantilever oscillation versus $\:{L}_{p}$. (**d**) Experimental and simulated frequency of cantilever oscillation showing a nonlinear decrease for increasing $\:{L}_{p}$.

Figs. 4(a, b) show the stiffness and damping coefficients extracted from step‑response experiments as a function of the piezoelectric‑layer length $\:{L}_{p}$. Typical step-response characteristics are shown in Figs. S1 and S2 in the Supplementary Information. The damping forces comprise different damping effects including intrinsic material-dependent dissipation and viscous air damping. Electromechanical coupling effects do not enter, as the step responses are conducted under short-circuit condition. The piezoelectric layer length affects the elastic and damping forces. As $\:{L}_{p}$ increases, both stiffness and damping increase. The stiffness rises with $\:{L}_{p}$ for a fixed total cantilever length of 10 mm, because a longer piezoelectric segment leaves a shorter proportion of the compliant cantilever material and, thus, increases the overall flexural rigidity. In particular, the damping coefficients exhibit a nonlinear increase, indicating that the structural loss becomes increasingly dominant for longer piezoelectric sections. Fig. S4 in the Supplementary Information shows the LEM simulation results for the average mechanical power during resonant self-actuation, along with the corresponding contributions of dissipated power as a function of $\:{L}_{p}$. For $\:{L}_{p}$ of 7 mm, more than 90% of the input energy is dissipated through structural damping. As the damping contribution decreases with decreasing $\:{L}_{p}$, structural damping accounts forapproximately 50% of the dissipation at $\:{L}_{p}$ of 5 mm and about 10% at $\:{L}_{p}$ of 3 mm. The remaining kinetic energy is primarily dissipated upon impact with the magnet, while only a small fraction is consumed by load-dependent electrical damping. The required tip mass increases with the piezoelectric length (see Table 1), underscoring the need to balance the enhanced dissipative forces with additional inertia to maintain a stable and continuous oscillatory motion.

The fabricated P-TM generator devices exhibit stable, resonant self-actuation near the source temperature $\:{T}_{source}$ of 100 °C. Under these conditions, Figs. 4(c) and (d) show experimental and simulated stroke and frequency values, respectively. As $\:{L}_{p}$ decreases and cycling frequency increases, the average heat intake as well as the heat dissipation via forced convection increase, as will be discussed in the following. The increasing cycling frequency causes an increase of the mean mechanical power delivered by the magnetic interaction, which is shown in Fig. S4 in the Supplementary Information.

The mechanical-to-electrical energy conversion of a piezoelectric structure is characterized by the effective electromechanical coupling coefficient $\:{k}_{eff}^{2}=\:\frac{{c}_{i}^{2}}{{C}_{0}k\:+\:{c}_{i}^{2}}$, whereby $\:{c}_{i}$ is the so-called force factor, $\:{C}_{0}$ is the capacitance and *\:k* the effective stiffness^34^. During oscillation, energy is exchanged between elastic energy, kinetic and electrical energy. The effective electromechanical coupling coefficient $\:{k}_{eff}^{2}\:$quantifies the ratio of converted energy and total (electrical and elastic) energy. The capacitance $\:{C}_{0}$ is estimated using an inductance-capacitance-resistance (LCR) meter, at frequencies far above the frequencies of cantilever oscillation given in Table S2 in the Supplementary Information. The coupling coefficients $\:{k}_{eff}^{2}$ reach 2.35% and 2.22% for P‑TM generators with a piezoelectric length $\:{L}_{p}$ of 6 and 7 mm, respectively, which compares with soft-PZT-based unimorph devices^35,36^, and decrease to 0.27% for $\:{L}_{p}$=3 mm. The results on coupling coefficients $\:{k}_{eff}^{2}$ are summarized in Table 1. These results may be compared with simulated average mechanical power of the oscillator and the effect of the different damping mechanisms on power dissipation due to mechanical impact of the cantilever tip with the magnet, structural damping and electromechanical damping, as shown in Fig. S4 in the Supplementary Information. The LEM simulations indicate that the contribution of electromechanical damping to overall damping increases from 0.2% ($\:{L}_{p}$=3 mm) up to 12% ($\:{L}_{p}$=7 mm) indicating a strong increase of power conversion for increasing $\:{L}_{p}$.

**Fig. 5:**
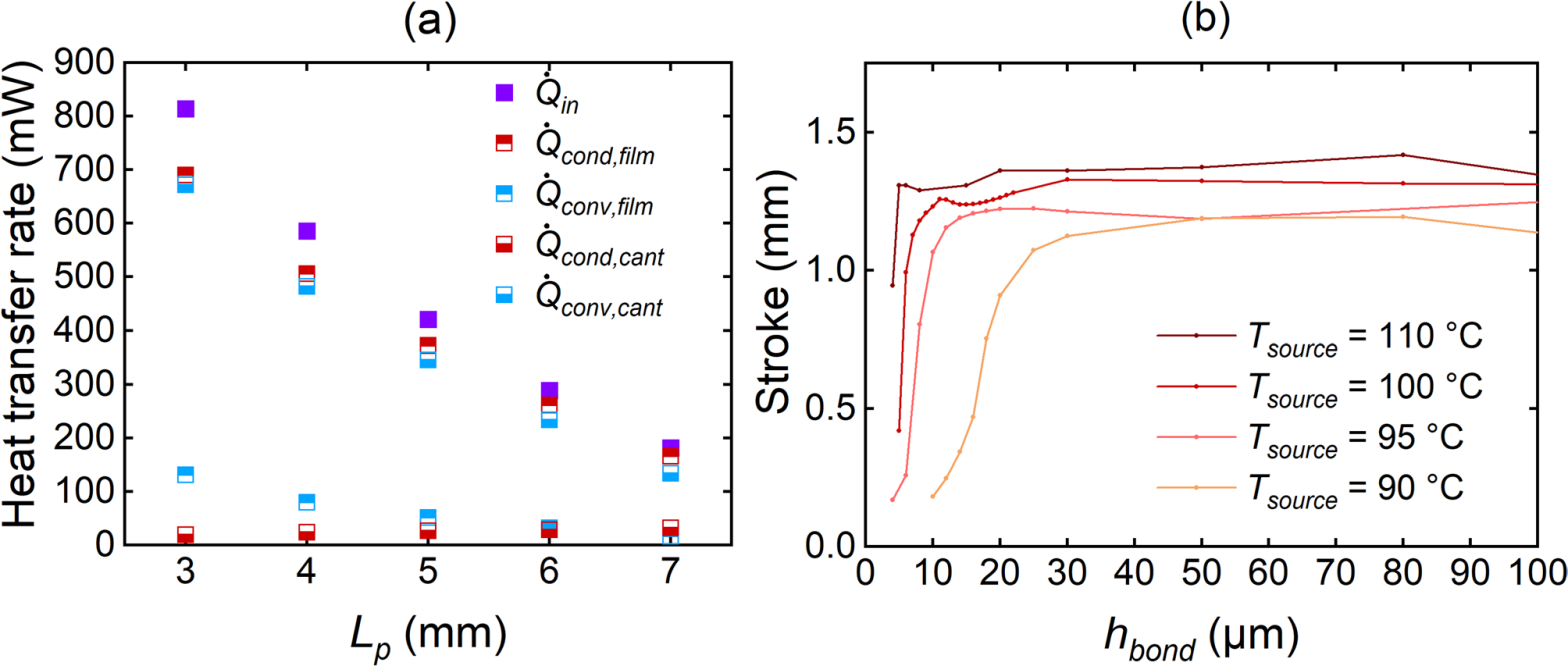
LEM simulation of heat transfer rates and oscillation stroke. (**a**) Average heat intake and dissipation versus length of the piezoelectric layer $\:{L}_{p}$ of a P‑TM generator with a tip mass of 80 mg at a source temperature of 100 °C. Legend: $\:{\dot{Q}}_{in}$ – average heat intake, $\:{\dot{Q}}_{cond,film}$ – heat transfer rate by conduction via the TM layer to the cantilever, $\:{\dot{Q}}_{conv,film}$ - heat dissipation rate by convection via the TM layer, $\:{\dot{Q}}_{cond,cant}$ - heat dissipation rate by conduction via the cantilever to the substrate, $\:{\dot{Q}}_{conv,cant}$ - heat dissipation rate by convection via the cantilever and piezoelectric layer. (**b**) LEM simulation of oscillation stroke versus bonding layer thickness $\:{h}_{bond}$ of a P‑TM generator with $\:{L}_{p}$ of 7 mm and a tip mass of 80 mg for different heat source temperatures as indicated. Below a critical layer thickness, the oscillation stroke drops abruptly because the TM layer is thermally short-circuited to the cantilever.

Tip mass and cantilever stiffness serve as a tuning parameter for resonant self‑actuation, yet the operating frequency $\:{f}_{op}$ also depends crucially on heat intake and heat dissipation. In the design, heat intake is optimized through the contact area between TM layer and heated magnet and minimization of roughness of the contacting surfaces. Figure 5(a) summarizes the simulated average heat intake and dissipation versus length of the piezoelectric layer $\:{L}_{p}$ of a P‑TM generator operating in resonant self-actuation mode. The heat intake increases for decreasing $\:{L}_{p}$ following the trend of increasing oscillation frequency and decreasing damping. In the present case, the heat intake increases from 180 to 800 mW for decreasing $\:{L}_{p}$ from 7 to 3 mm, respectively. At the same time, conductive and convective heat transfer through the TM layer increase, whereby heat conduction via the bonding layer to the cantilever is the dominant mechanism.

The heat transfer to the cantilever depends on the thermal resistivity of the TM layer’s bonding layer and the temperature difference between TM layer and cantilever. For efficient operation the bonding layer’s thermal resistance must exceed a critical threshold to prevent thermal short-circuiting^37^. This is demonstrated in Fig. 5(b) for a P-TM generator with $\:{L}_{p}$ = 7 mm and a tip mass *\:m* = 80 mg. For decreasing bonding layer thickness, the actuation stroke exhibits an abrupt drop below a critical value, because the input thermal energy dissipates too quickly to cause a suitable change in magnetic force. Therefore, tuning of thermal resistances is required to control heat dissipation.

Owing to the small cross-section of the cantilever, the thermal resistance of the cantilever limits the maximum heat flow that can be dissipated by conduction to the substrate. Assuming a source temperature of 100 °C, a substrate temperature of 22 °C and a thermal resistance of about $\:{R}_{cant}$, 1970 KW^− 1^ at $\:{L}_{p}$ of 7 mm, the maximum heat flow to the substrate is only about 40 mW. This value further decreases for increasing $\:{L}_{p}$ as heat convection strongly increases for increasing frequency. Therefore, conduction to the substrate through the cantilever plays a minor role for heat dissipation. In this case, roughly 80% of heat intake must be rejected via forced convection to the surfaces of the cantilever and piezoelectric layer. Thereby, the low thermal resistance of the piezoelectric layer and its bonding layer, of below 16 KW^− 1^, does not impede heat transfer. The increase of forced convection with increasing oscillation frequency results in lowering the cantilever temperature and, in turn, allows for enhanced heat transfer from the TM layer to the cantilever. As damping adversely affects the oscillation frequency (Fig. 4b), low damping is desirable for efficient heat dissipation.

### Electrical power output

**Fig. 6:**
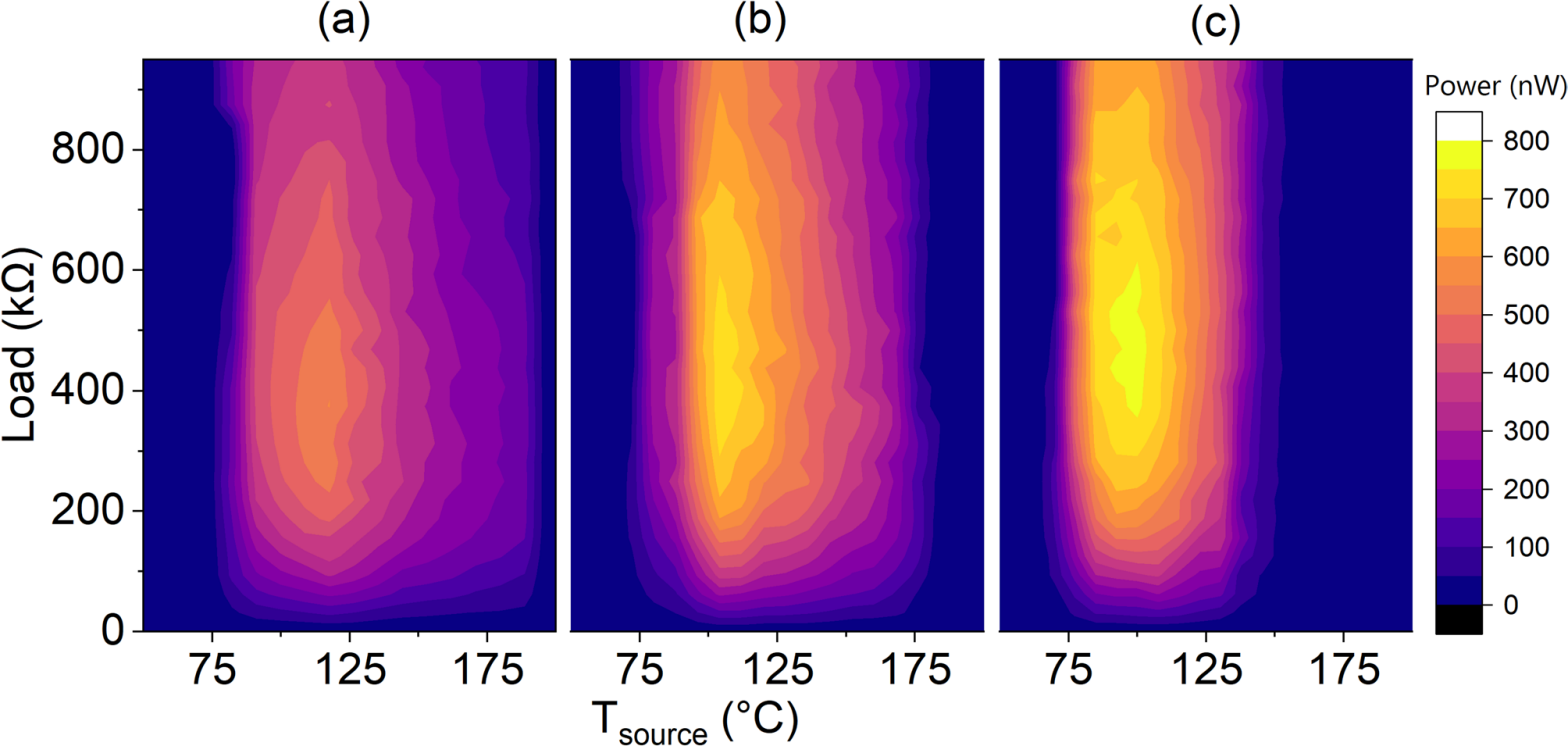
Experimentally determined electrical AC power output of the P-TM generators with respect to source temperature and load resistance for different lengths of piezoelectric layer $\:{L}_{p}$ of 3 mm (**a**), 5 mm (**b**) and 7 mm (**c**). The values given are root-mean-square values determined from load and current.

The mechanical oscillation of the P-TM generator is converted into an AC voltage signal with peak voltage up to 1.3 V. These voltage levels enable power rectification, as demonstrated in Fig. S5 in the Supplementary Information. Figure 6 summarizes the experimental results on average AC power output of the P-TM generators for different piezoelectric layer lengths $\:{L}_{p}$ of 3, 5 and 7 mm. The heat source temperature is varied in the range of 50–200 °C, while the load resistances cover the range up to 950 kΩ. For all P-TM generators, the peak power output occurs near a heat source temperature of 100 °C at a load resistance of 400 to 500 kΩ. The maximum AC power output increases with $\:{L}_{p}$ demonstrating enhanced energy conversion capability. Maximum AC power output of 0.8 µW is obtained for the device with largest $\:{L}_{p}$ of 7 mm, which corresponds to a power per device footprint of 4 µW/cm². At smaller lengths of the piezoelectric layer $\:{L}_{p}$ of 5 and 3 mm, the maximal AC power reaches 0.7 and 0.6 µW, respectively. The decrease in AC power output for decreasing $\:{L}_{p}$ correlates with a noticeable increase in the temperature bandwidth. While all TM generators start operating at a heat source temperature of around 75 °C, the maximum operational temperature decreases from about 180 °C for $\:{L}_{p}$ of 3 mm, to about 160 °C and 130 °C for $\:{L}_{p}$ of 5 and 7 mm, respectively. This indicates a trade-off between AC power output and operational temperature range.

## Discussion

We present a compact P-TM generator with a footprint of 10 × 2 mm² for conversion of low-grade waste heat at temperatures near 100 °C to electricity. The development of first demonstrators with multilayer design is presented including a piezoelectric layer of PZT and a TM layer of Ni-Mn-Ga with 30 μm and 20 μm thickness, respectively. The demonstrators are fabricated by hybrid integration of TM film and piezoelectric layer using standard bonding technologies. The coupled thermal-mechanical-electrical interaction between the different layers is investigated experimentally and by lumped-element simulations. In particular, the effects of tip mass and length of the piezoelectric layer as well as heat transfer and damping on resonant self-actuation are analyzed to elaborate design criteria for optimal power conversion.

Tip mass and cantilever stiffness are important tuning parameters to achieve resonant self‑actuation. At too small tip mass and high stiffness, the mechanical oscillation frequency is too high for sufficient heat transfer during a thermal cycle. Thus, several oscillations are required for the TM layer to cool in order to regain sufficient magnetic attraction and to sustain continuous oscillation. We present an experimental parameter study by changing the piezoelectric layer length $\:{L}_{p}$, while maintaining the overall cantilever length. Increasing the length of the piezoelectric layer causes an increase in cantilever effective stiffness and damping. The resulting change of frequency can be compensated by adjusting the tip mass. However, low damping is desirable for efficient heat transfer through convection, as damping adversely affects the oscillation frequency and stroke. A reduction in actuation stroke leads to an increase of minimal gap between TM layer and magnet during oscillation and, thus, hampers regaining magnetic attraction. Therefore, any change of actuation stroke requires adjustment of the distance between heated magnet and cantilever tip.

Resonant self-actuation relies on a careful synchronization of the oscillatory motion and thermal cycle of the TM layer: The magnetic attraction force needs to overcome damping forces so that the TM layer returns close to the magnet in each oscillation cycle. In addition, the heat intake at contact between TM layer and heated magnet needs to enable a sufficient temperature increase for the cantilever to retract, while heat dissipation must allow for sufficient temperature decrease of the TM layer to regain sufficient magnetic attraction in order to sustain continuous oscillation. The presented LEM simulations on time-dependent actuation stroke and frequency (Fig. 3) as well as the time-dependent AC power signals (Fig. S6) well describe the experimental results, which validates our LEM model.

The P-TM generators show a rather large temperature bandwidth as presented in Fig. 6. Thus, slight deviations in $\:{T}_{C}$ have no impact on performance, as long as the source temperature $\:{T}_{source}$ is not too close to $\:{T}_{C}$. The temperature bandwidth of resonant self-actuation is due to a self-tuning effect^14^. For increasing source temperature $\:{T}_{source}$, heat intake increases, which is compensated by increasing oscillation frequency and corresponding convective cooling. This self-tuning effect is observed until a critical maximum $\:{T}_{source}$, at which heat input can no longer be compensated by heat dissipation. In this case, heat intake and heat dissipation get out of balance resulting in an increase of the TM layer’s mean temperature, which in turn reduces the change of magnetic actuation force. At too low heat source temperatures, the heat intake is not enough to cause a sufficient change of magnetic attraction force. Both effects give rise to an optimal temperature window of resonant self-actuation. The different temperature regimes are accurately described by the LEM simulations. However, the model is not intended to predict the full nonlinear dynamic transitions between the different regimes, which may occur rather abruptly depending on the TM material (maximum magnetization, $\:\varDelta\:M$/$\:\varDelta\:T$) and system parameters.

In the present design, forced heat convection via the surfaces of cantilever and piezoelectric layer is the dominant heat dissipation mechanism. The presented LEM simulations show that the bonding layer between piezoelectric layer and cantilever, as well as the piezoelectric layer itself, have only a minor effect on convective heat transfer and, thus, favor heat dissipation. Further improving the thermal conductivity of the cantilever is desirable to improve heat transfer through to the substrate and to further enhance thermomagnetic cycle frequency.

Both, heat intake and heat losses determine the maximum temperature change of the TM layer. For the investigated P-TM generator with two stacked Ni-Mn-Ga layers of 10 μm thickness and a heat source temperature of 100 °C, the TM layer temperature varies between about 99 and 86 °C. This temperature change decreases for decreasing heat source temperature. Below 75 °C, it becomes too small to enable a sufficient change of magnetic attraction required for resonant self-actuation.

The presented P-TM generators produce a maximum AC power output near a heat source temperature of 100 °C at a load resistance in the range of 400–500 kΩ. The maximum AC power output increases with the length of piezoelectric layer $\:{L}_{p}$ despite the decrease in heat intake (Fig. 5) and resulting decrease in frequency and stroke of resonant self-actuation. However, this decrease is compensated by the strong increase of electromechanical coupling between cantilever and piezoelectric layer. Our analysis of damping mechanisms presented in Fig. S4 in the Supplementary Information reveals a strong increase of electromechanical damping from 0.2% to 12% indicating a strong increase of power conversion for increasing $\:{L}_{p}$. The AC power reaches a maximum of 800 nW at $\:{L}_{p}$ of 7 mm, which corresponds to a maximal AC power per device footprint of 4 µW/cm². At the same time, the temperature window of resonant self-actuation decreases from 75° − 180 °C for $\:{L}_{p}$ = 3 mm to 75°-130 °C for $\:{L}_{p}$ = 7 mm indicating a trade-off between operational temperature range and AC power output. At small $\:{L}_{p}$, the damping is rather low (Fig. 4) and, thus, the P-TM generator can sustain oscillation at lower magnetic forces occurring at larger operation temperatures. In addition, the higher frequency at small $\:{L}_{p}$ allows to dissipate more heat input through enhanced heat convection, which enables resonant self-actuation at higher heat source temperatures. On the other hand, electromechanical coupling strongly decreases at small $\:{L}_{p}$ resulting in reduced power output.

The piezoelectric layer converts the mechanical energy to electrical energy at a sufficiently high voltage level of 1.3 V AC. The presented approach of combing TM film-induced resonant self-actuation with piezoelectric conversion facilitates voltage rectification opening up a route to generate usable DC power from low-grade waste heat. After rectification, this corresponds to 1.25 µW/cm² of DC power at a voltage of 2 V DC, as demonstrated in Fig. S5 in the Supplementary Information.

When comparing the power densities with TEG devices, the temperature difference and length scale should be taken into account, as it defines the applicability and competitiveness^12^. The AC power output of the P-TM generator compares well with state-of-the art TEG devices at the mm-length scale, if the size of the heat sink is taken into account. This is due to limitations in microfabrication of TEG devices, which strongly limits $\:\varDelta\:T$ requiring large heat sinks exceeding the size of the TEG module^1^. This is confirmed by a recent investigation on TEG devices for body temperature harvesting achieving a power density of 7 µW/cm² at ΔT of 7 K when using a plate fin sink for heat dissipation through natural convection^38^.

## Conclusions

This paper presents the development of a compact generator for conversion of low-grade waste heat at temperatures near 100 °C to electricity. Thermal energy is converted in two steps comprising resonant self-actuation of an elastic cantilever using the large temperature-induced change of magnetization at the ferromagnetic transition of a TM film of Ni-Mn-Ga and subsequent piezoelectric conversion of mechanical energy of the oscillator into electrical energy using a PZT layer. The performance of the generator is characterized experimentally and by LEM simulations to elucidate the effects of tip mass and length of the piezoelectric layer as well as heat transfer and damping. Tip mass and cantilever stiffness serve as a tuning parameter for the frequency of resonant self‑actuation. In addition, heat intake and heat dissipation have to be matched, which is achieved by optimizing the heat transfer at contact to the heat source (heated magnet) and tuning the thermal resistivity of the TM layer’s bonding layer as well as by tuning the frequency. Thereby, the temperature change of the TM layer determines the change in magnetic attraction required to overcome damping and to sustain continuous oscillation. The peak voltage generated by piezoelectric conversion enables rectification using standard electrical circuitry. In optimal case, a single P-TM generator generates an average AC power output of 4 µW/cm² allowing for 1.25 µW/cm² of DC power after rectification. As the TM film is exposed to low strain and small $\:\varDelta\:T$ at the cantilever, long lifetime is expected. This has been confirmed by running the P-TM generators for three days without any degradation corresponding to more than 5 million cycles. As the Curie temperature of PZT material of 180 °C is well above the temperature of the cantilever during resonant self-actuation, no depolarization effects have been observed. The results indicate that further improvement of material properties and design, e.g. increasing the thermal conductivity of the cantilever and tuning the intrinsic damping and thickness of the piezoelectric layer, will enable further enhancement of energy conversion and optimization of power output. From our analysis we also infer that the current thickness of the piezoelectric layer of 30 μm should be further reduced to improve coupling between cantilever and piezoelectric layer at large $\:{L}_{p}$ to enhance the intake of mechanical power and further optimize power output. Concerning material strategies, the use of piezoelectric material with high Q-factor is beneficial.

This investigation is based on Ni-Mn-Ga films with Curie temperature $\:{T}_{c}$ of 80 °C allowing for resonant self-actuation at about 100 °C. The presented results and insights may well be transferred to other high-performance TM layers with large temperature-dependent change in magnetization within a narrow temperature interval $\:\varDelta\:M/\varDelta\:T$ near room temperature. Upscaling the number of oscillators to oscillator arrays with corresponding power conditioning circuitry will allow increasing the overall power output and, thus, bridging the gap toward applications like, e.g., autonomous sensor systems and low-power electronic devices. Currently, fabrication tolerances of fabrication at lab scale are a major limitation in upscaling. Regarding thermal management, precise alignment of the cantilevers with respect to the heat source poses a challenge on fabrication technology. Regarding electrical integration, rectification of the output of the oscillator array requires either synchronous operation of all oscillating cantilevers or rectification of the AC voltage signals of each oscillating cantilever.

## Methods

### Experimental

A high temperature permanent magnet of Samarium Cobalt (SmCo) with operating temperature tolerance up to 350 °C is used as the magnetic field and heat source. It has a cross-sectional area of 3 × 3 mm² and an axially pole distance of 8 mm. The magnet is positioned with respect to the Ni-Mn-Ga film surface at an optimum distance and angle using a digital microscope to ensure maximum contact but to avoid impact to the magnet surface under operation. The magnet is heated until a stationary temperature is reached before experiments are started. The heated magnet can be translated and rotated using motorized stages, enabling controlled alignment with respect to the cantilever front. A laser position sensor is used to determine the displacement of the cantilever tip, while current and voltage amplifiers are used to measure the electrical signals across the load resistor. The analog signals are recorded using a 32-bit data acquisition unit at a sample rate of 15 kS/s. Afterwards, a mean filter of 5 samples and a stride of 5 is applied, to down sample and filter the signals. The variable load allows selection between 16 known resistance values in a range of 0 and 1 MΩ. Root-mean-square (RMS) power is calculated using the current signal and the selected load value. The thermal energy harvesting performance is characterized with respect to source temperature under quasistatic conditions by ramping the magnet temperature at a rate of approximately 2 K/min from 60 °C to 180 °C. At the same time, the load resistance is continuously stepped through its defined values while displacement, voltage, and current are recorded in 3 s intervals. Power is calculated for each load-temperature pair and linearly interpolated between points to generate the colored power-temperature-load plots shown in Fig. 6. Effective stiffness, damping and piezoelectric coupling coefficients are derived from experimental step response tests with short-circuited electrical terminals. Step response is performed on the same setup by deflecting and releasing the cantilever through specific magnet movements. From the measured step response, the effective mechanical parameters are derived as follows. Parameters $k = \left( {2\pi f_{0} } \right)^{2} m$ and $\:{d}_{1}=2mD$ are determined using the tip mass *\:m*, eigenfrequency $\:{f}_{0}$ and decay rate *\:D* of the step response’s oscillatory decay. Tip mass is measured using a precision scale. The fit function

1 $$\:g\left(t\right)=A\cdot\:\mathrm{s}\mathrm{i}\mathrm{n}\left(2\pi\:ft+\phi\:\right)\cdot\:\mathrm{exp}\left(-Dt\right)+a$$

    

is used to determine eigenfrequency and decay rate of the first three oscillatory cycles. Parameter $\:{d}_{2}$ is adjusted to match the first positive peak amplitude of simulated and experimental step responses. Typical step responses are shown in Fig. S1 in the Supplementary Information. Force factors $\:{c}_{i}=I/\left(2\pi\:fX\right)$ are determined by using the fit function (Eq. (1)) to current and displacement signals of the step response, which is shown in Fig. S2 in the Supplementary Information. Values *\:I* and *\:X* are the current and displacement amplitudes (*\:A*) of the fit function. The force factors based on the experiments on Players of thickness 30 μm compare well with simulated force factors (FEM, Comsol Multiphysics) as shown in Fig. S3 in the Supplementary Information using datasheet values of corresponding bulk piezoelectric materials indicating that mechanical thinning did not affect the piezoelectric properties.

### Lumped element model

**Fig. 7:**
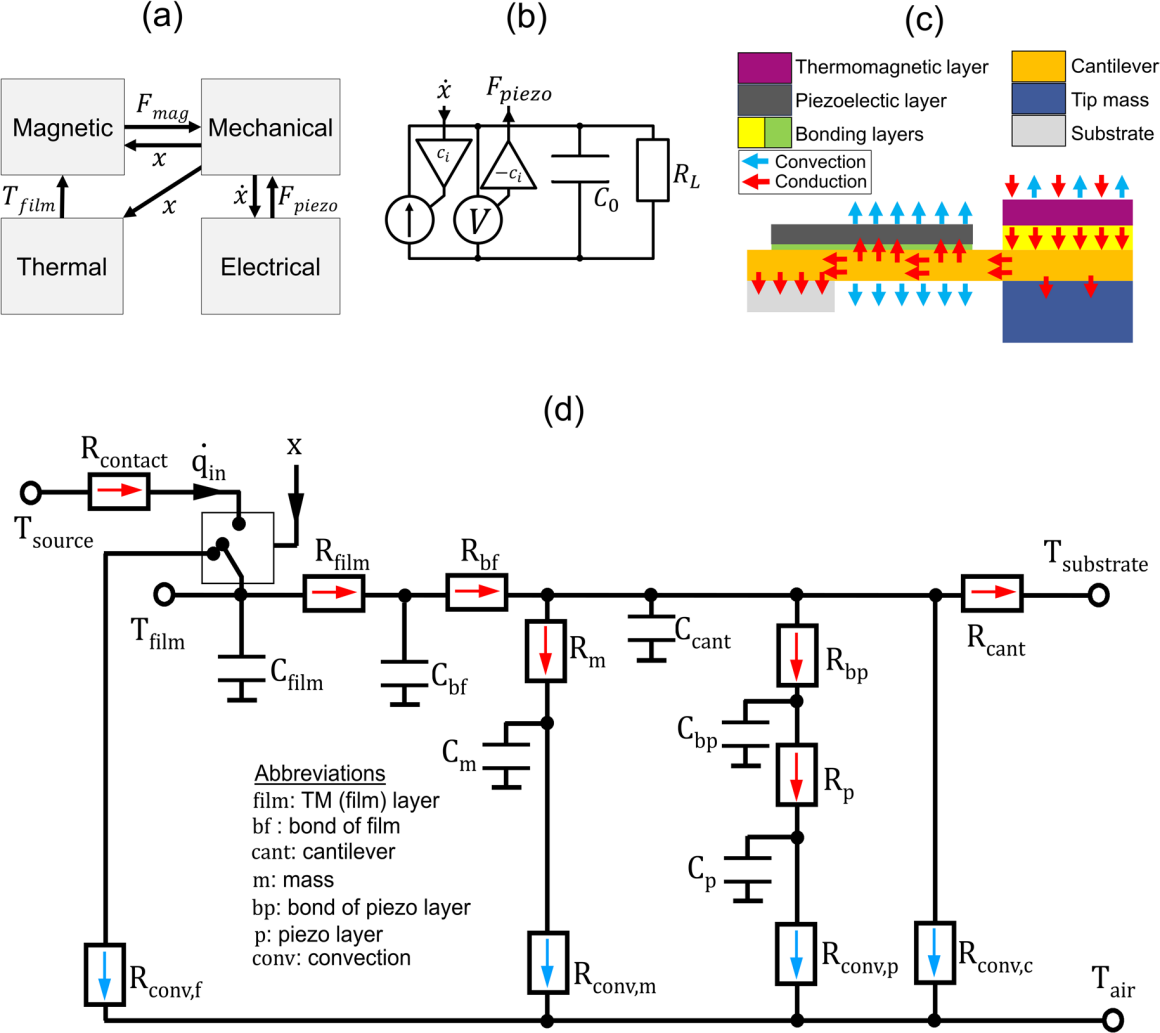
Schematic overview of the LEM. (**a**) Coupling between each physical sub-model. The magnetic sub-model is represented by a lookup table that returns the magnetic force as a function of distance between TM layer and heated magnet and temperature of the TM layer. The mechanical dynamics are described by Eq. (2). (**b**) Equivalent circuit used in the electrical sub-model to describe the piezoelectric coupling by the force factor $\:{c}_{i}$, a speed ($\:\dot{x}$) controlled current source and a voltage meter, which feeds back the counteracting force $\:{F}_{piezo}$. $\:{C}_{0}$ represents the capacitance of the piezoelectric layer and $\:{R}_{L}$ the load resistor. (**c**) Schematic diagram illustrating the heat intake, conduction and convection paths in the P-TM generator. In the thermal sub-model, each component is represented by a thermal mass and thermal resistivity, forming a thermal network. (**d**) Corresponding thermal network used in the thermal sub-model. The red and blue arrows indicate the effective conductive and convective heat transfer directions. At the top left of the thermal network, a position $\:x\:$– controlled switch is used to model the heat transfer from the heat source to the TM layer through the contact resistance $\:{R}_{contact}$. When not in contact with the heat source, the switch connects the TM layer to an ambient temperature reservoir via $\:{R}_{conv,f}$, modeling convective heat dissipation of the TM layer.

The model is divided into magnetic, mechanical, thermal and electrical sub-models that are linked via scalar state variables, as shown in Fig. 7(a). The magnetic sub-model considers experimentally determined magnetic attraction forces $\:{F}_{mag}$ between a Ni-Mn-Ga film sample and the permanent magnet as a function of film distance and film temperature $\:{T}_{film}$, which are listed in a lookup table. The distance- and temperature-dependent characteristics of magnetic attraction force are presented in Fig. S7 in the Supplementary Information. The efficiency of TM conversion is given by the ratio of magnetic energy $\:{E}_{mag}$ and input thermal energy $\:{Q}_{in}$ ($\:{\eta\:}_{TM}={E}_{mag}/{Q}_{in}$) per cycle. The output magnetic energy $\:{E}_{mag}$ is obtained by calculating the enclosed area of the TM cycle, while $\:{Q}_{in}$ is estimated by thermal LEM simulations as described below. A typical TM cycle is shown in Fig. S8 for the case of $\:{L}_{p}$=7 mm in the Supplementary Information. The resulting efficiencies are summarized for the different $\:{L}_{p}$ in Table 1.

The mechanical sub-model considers the one-dimensional equation of motion

2 $$\:m\ddot{x}+{d}_{1}\dot{x}+{d}_{2}\dot{x}\left|\dot{x}\right|+kx={F}_{contact}+{F}_{piezo}+{F}_{mag}$$

approximating the motion of the tip mass *\:m*, that is attached to the cantilever with stiffness *\:k*. Damping forces are described by the damping coefficients $\:{d}_{1}$ and $\:{d}_{2}$, whereby the quadratic damping coefficient $\:{d}_{2}$ is used to better describe the strong damping effects introduced by the PZT material leading to a non-linear step response that cannot be adequately described by viscous damping ($\:{d}_{1}$) alone, which is demonstrated in Fig. S1 in the Supplementary Information. The force at the rigid contact between cantilever front and magnet, $\:{F}_{contact}$, is modeled by a large stiffness and additional damping coefficient that engage only during contact. The piezoelectric reaction force $\:{F}_{piezo}$ is part of the electromechanical coupling, which is determined in the electrical sub-model. Figure 7(b) shows the circuit in the electrical sub-model including the capacitance of the piezoelectric layer and the load resistor. Here, the linear electromechanical coupling is modeled using the force factor $\:{c}_{i}$, a speed controlled current source and voltage meter.

The thermal sub-model describes the heat intake, heat flow and heat dissipation of the P‑TM generator, which is illustrated in Fig. 7(c). The heat intake from heat source (heated magnet) to TM layer depends on the heat transfer coefficient at contact. Heat dissipation occurs via heat transfer through the non-conductive bonding layer, which is a non-conductive epoxy. Changing the thickness of the bonding layer changes the thermal resistance between TM layer and cantilever. Thus, the heat flow to the cantilever can be controlled by changing the thickness of the bonding layer, which is an important parameter to control the balance of heat intake and dissipation. Heat is dissipated by heat conduction along the cantilever and heat convection mainly via the surfaces of the cantilever and piezoelectric layer, whereby the convective heat transfer coefficient largely depends on the oscillation frequency. Each component of the P‑TM generator, such as the TM layer, bonding layers, cantilever, and piezoelectric layer, is represented by a thermal capacitance and a thermal resistance that model its thermal mass and thermal resistivity, respectively^39^. The thermal resistance for conduction through a component is expressed as $\:{R}_{cond}=\frac{l}{{k}_{cond}\cdot\:\:{A}_{cond}}$ where *\:l* is the conduction length, $\:{A}_{cond}$ the cross‑sectional area for conduction, and $\:{k}_{cond}$ the material’s thermal conductivity. In the resulting thermal network (Fig. 7(d)) thermal energy is transferred from nodes with higher temperature to nodes with lower temperature. The absolute temperature in Kelvin of a component is obtained from $\:T\:=\frac{q}{{C}_{th}}$ with *\:q* denoting the stored thermal energy and $\:{C}_{th}$ the thermal capacitance.

The heat transfer from the magnet to the TM layer is modeled using a position-controlled switch inside the thermal network, as seen on the top left side of Fig. 7(d). When the TM layer contacts the magnet, the switch in the thermal network connects $\:{C}_{film}$ to the heat reservoir at a constant temperature $\:{T}_{source}$ via the contact resistance $\:{R}_{contact}$. Consequently, the TM layer’s temperature raises as heat is absorbed. The effective contact time is estimated based on the duration during which the cantilever front remains within the gap between cantilever front and magnet of about 0.2 mm, assuming that heat intake occurs not only through direct solid-solid contact but also through hot air within this gap. At a heat source temperature of 100 °C, this estimate gives rise to effective heat transfer coefficients and input thermal energies per cycle $\:{Q}_{in}$ that are summarized in Table 1 versus $\:{L}_{p}$.

In the absence of contact, a portion of the heat in the TM layer conducts to the cantilever through the bonding layer, while the other part is dissipated by convection. Convection occurs when air molecules in the vicinity exchange thermal energy with the surface. Because the cantilever is at a higher temperature than the surrounding air, thermal energy from the cantilever is transferred to the gas molecules. The oscillatory motion of the cantilever continuously renews the layer of cool air in contact with the surface through forced convection, thereby augmenting the overall heat-transfer rate. Larger oscillation amplitudes and higher frequencies increase the induced air flow and, consequently, enhance the effective convective heat-transfer. Accurately predicting this effect requires a modeling approach that accounts for the coupled fluid-dynamic and thermal interactions of the oscillating cantilever and the surrounding air. In the LEM approach used here, this effect is approximated using the thermal resistance $\:{R}_{conv}=\frac{1}{{A}_{conv}\cdot\:\:{h}_{conv}}$, which enables heat transfer to the ambient temperature reservoir. Thereby, $\:{A}_{conv}$ is the surface area exposed to convection and $\:{h}_{conv}$ the effective convective heat‑transfer coefficient, which correlates with the oscillation speed, as shown in Fig. S9 in the Supplementary Information. The LEM is implemented in Simulink with the Simscape toolbox (MATLAB R2024b). Through the thermal network used in the model, the heat flow path can be distinguished and the temperature change experienced by each section can be determined depending on its dimensions and thermal properties. The modelling parameters are summarized in Table 1 together with the calculated efficiency of TM conversion and the effective electromechanical coupling coefficient of the presented P-TM generators. The rather low efficiencies are due to the rapid oscillation under the dynamic conditions of resonant self-actuation being far from stationary conditions and associated with small ΔT and small change of magnetization $\:\varDelta\:M$. Thus, these conditions are optimized for electrical power output instead of optimal efficiency.

Further information on experimental and simulation procedures are available in the Supplementary Information.

**Table 1:** Summary of the parameters used for LEM simulations and parameters of individual P-TM generators. Legend: $\:{L}_{p}$ *–* Length of the piezoelectric layer, *\:m* – tip mass, *\:k* – effective stiffness, $\:{d}_{1}$ – viscous damping coefficient, $\:{d}_{2}$ – quadratic damping coefficient, $\:{c}_{i}$ – force factor, $\:{C}_{0}$ – capacitance of piezo element, $\:{h}_{conv}$ – convection coefficient, $\:{h}_{cont}$ – heat transfer coefficients at contact, $\:{E}_{mag}$ *–* magnetic energy per cycle, $\:{Q}_{in}$ – input thermal energy per cycle, $\:{\eta\:}_{TM}$ – efficiency of TM conversion, $\:{k}_{eff}^{2}$ – effective electro-mechanical coupling coefficient.

Parameters used by all device variants								Reference
TM layer width and length					[mm]		2	This work
TM layer thickness					[µm]		20	This work
TM layer density					[kg m^− 3^]		8020	^39^
TM layer thermal conductivity					[W m^− 1^ K^− 1^]		23.2	^39^
TM layer specific heat capacity					[J kg^− 1^ K^− 1^]		490	^39^
TM bonding layer width and length					[mm]		2	This work
TM bonding layer thickness					[µm]		30	This work
TM bonding layer density					[kg m^− 3^]		1250	^39^
TM bonding layer conductivity					[W m^− 1^ K^− 1^]		0.33	^39^
TM bonding layer specific heat capacity					[J kg^− 1^ K^− 1^]		2100	^39^
Cantilever length					[mm]		10	This work
Cantilever width					[mm]		2	This work
Cantilever thickness					[µm]		20	This work
Cantilever density					[kg m^− 3^]		8500	^39^
Cantilever thermal conductivity					[W m^− 1^ K^− 1^]		109	^39^
Cantilever specific heat capacity					[J kg^− 1^ K^− 1^]		400	^39^
Piezoelectric layer width					[mm]		2	This work
Piezoelectric layer thickness					[µm]		30	This work
Piezoelectric layer density					[kg m^− 3^]		8100	Data sheet
Piezoelectric layer thermal conductivity					[W m^− 1^ K^− 1^]		1.2	Data sheet
Piezoelectric layer specific heat capacity					[J kg^− 1^ K^− 1^]		380	Data sheet
Piezoelectric bonding layer width					[mm]		2	This work
Piezoelectric bonding layer thickness					[µm]		15	This work
Piezoelectric bonding layer density					[kg m^− 3^]		3500	^40^
Piezoelectric bonding layer thermal conductivity					[W m^− 1^ K^− 1^]		29	Data sheet
Piezoelectric bonding layer specific heat capacity					[J kg^− 1^ K^− 1^]		173	^40^
Thermal contact area					[mm²]		4	This work
Contact stiffness					[N m^− 1^]		1ꞏ10^12^	This work
Contact damping coefficient					[N m^− 1^ s]		1ꞏ10^4^	This work
Source temperature $\:{T}_{source}$					[K]		373	This work
Air temperature $\:{T}_{air}$					[K]		295	This work
Substrate temperature $\:{T}_{substrate}$					[K]		295	This work
Load resistance $\:{R}_{load}$					[kΩ]		500	This work

Parameters of individual P-TM generators								Reference
$\:{L}_{p}$	[mm]	3	4	5		6	7	This work
*\:m*	[mg]	7	20	30		40	80	This work
*\:k*	[N m^− 1^]	0.84	1.63	2.06		2.09	3.28	This work
$\:{d}_{1}$	[µN s m^-^^1^]	195	195	343		634	1291	This work
$\:{d}_{2}$	[µN s^2^ m^-2^]	100	500	800		1000	2000	This work
$\:{c}_{i}$	[µC m^− 1^]	4	12	15		23	31	This work
$\:{C}_{0}$	[nF]	7.12	8.17	9.36		10.50	12.93	This work
$\:{h}_{conv}$	[W m^− 2^ K^− 1^]	560	320	200		125	64	This work
$\:{h}_{cont}$	[W m^− 2^ K^− 1^]	16.1	14,7	17.7		13.3	7.3	This work
$\:{E}_{mag}$	[µJ]	4.8	4.8	3.7		2.7	0.7	This work
$\:{Q}_{in}$	[mJ]	18.2	13.8	11.4		10.1	8.0	This work
$\:{\eta\:}_{TM}$	[%]	0.026	0.035	0.033		0.026	0.008	This work
$\:{k}_{eff}^{2}$	[%]	0.27	1.07	1.15		2.35	2.22	This work

## Supplementary Information

Below is the link to the electronic supplementary material.

Supplementary Material

## Data Availability

The datasets generated during and/or analyzed during the current study are available from the corresponding author on reasonable request.
