# Supplementary material for: A miniature piezoelectric-thermomagnetic generator for low-grade waste heat recovery
Source: Sci Rep. 2026 Jul 31;16:23717. doi: 10.1038/s41598-026-59705-2 (PMC13427754; doi:10.1038/s41598-026-59705-2)
Supplement: Supplementary file 1 — Supplementary Material [file 41598_2026_59705_MOESM1_ESM.docx]

# Supplementary Information

## A Miniature Piezoelectric-Thermomagnetic Generator for Low-Grade Waste Heat Recovery

Maxim Wischnewski^1^, Joel Joseph^1^, Makoto Ohtsuka^2^, Hiroyuki Miki^3^, Manfred Kohl^1,*^

^1^ Institute of Microstructure Technology, Karlsruhe Institute of Technology (KIT), Karlsruhe, Germany

^2^ Institute of Multidisciplinary Research for Advanced Materials, Tohoku University, Sendai, Japan

^3^ Faculty of Science and Engineering, Ishinomaki Senshu University, Miyagi, Japan;

*****Corresponding Author: [manfred.kohl@kit.edu](mailto:manfred.kohl@kit.edu)

Author information:

Maxim Wischnews ([maxim.wischnewski@kit.edu](mailto:maxim.wischnewski@kit.edu))

Joel Joseph ([joel.jospeh@kit.edu](mailto:joel.jospeh@kit.edu))

Makoto Ohtsuka ([makoto.ohtsuka.d7@tohoku.ac.jp](mailto:makoto.ohtsuka.d7@tohoku.ac.jp))

Hiroyuki Miki ([hiroyuki.miki.k6@isenshu-u.ac.jp](mailto:hiroyuki.miki.k6@isenshu-u.ac.jp))

Manfred Kohl ([manfred.kohl@kit.edu](mailto:manfred.kohl@kit.edu))

### A.1 Geometric Parameters

**Table S1:** Summary of geometric parameters of the P-TM generators measured by digital microscope and height measurement gauge. $\boldsymbol{L}_{\boldsymbol{tot}}$**:** total cantilever length; $\boldsymbol{L}_{\boldsymbol{p}}$**:** nominal and actual length of piezoelectric layer; $\boldsymbol{w}_{\boldsymbol{p}}$**:** width of piezoelectric layer; $\boldsymbol{t}_{\boldsymbol{p}}$**:** thickness of piezoelectric layer; $\boldsymbol{t}_{\boldsymbol{b}}$**:** bonding layer thickness; $\boldsymbol{w}_{\boldsymbol{e}}$**:** width of electrode.

| $\boldsymbol{L}_{\boldsymbol{tot}}$ **(mm)** | $\boldsymbol{L}_{\boldsymbol{p}}$ **(mm)** | $\boldsymbol{w}_{\boldsymbol{p}}$ **(mm)** | $\boldsymbol{t}_{\boldsymbol{p}}$ **(µm)** | $\boldsymbol{t}_{\boldsymbol{b}}$ **(µm)** | $\boldsymbol{w}_{\boldsymbol{e}}$ **(mm)** |
| --- | --- | --- | --- | --- | --- |
| 10.27 | (7) 6.90 | 2.24 | 24 ± 3 | 15 ± 2 | 1.7 |
| 9.95 | (6) 5.83 | 2.16 | 23 ± 3 | 15 ± 2 | 1.7 |
| 9.65 | (5) 4.75 | 1.96 | 25 ± 3 | 15 ± 2 | 1.7 |
| 9.88 | (4) 3.80 | 1.96 | 27 ± 3 | 15 ± 2 | 1.6 |
| 9.92 | (3) 2.96 | 2.10 | 26 ± 3 | 15 ± 2 | 1.7 |

### A.2 Step response characterization

| 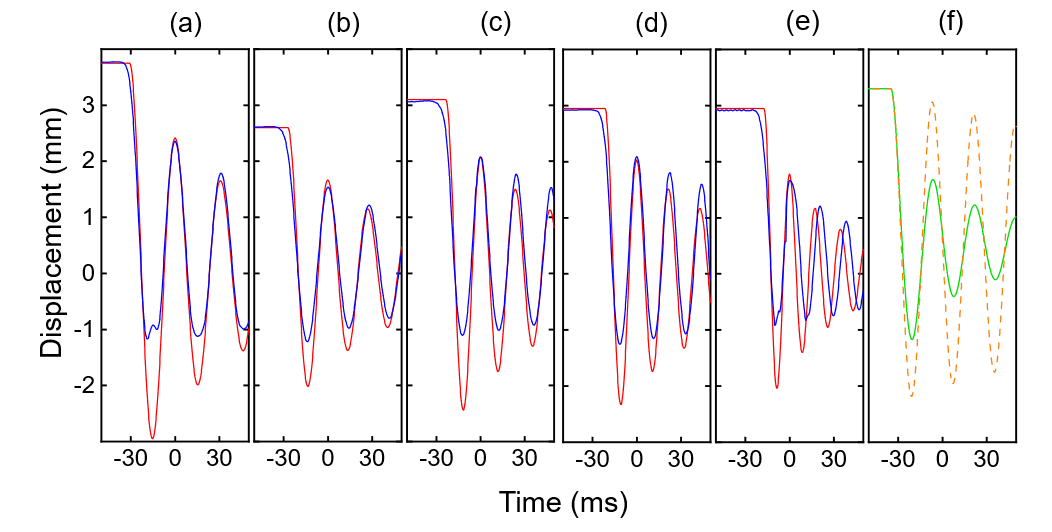 |
| --- |
| **Figure S1:** Experimental (blue) and simulated (red) step responses of cantilever devices for different length of the piezoelectric layer $L_{p}$ of (a) $L_{p}$ = 7 mm, $m$ = 80mg, (b) $L_{p}$ = 6 mm, $m$ = 40 mg (c) $L_{p}$ = 5 mm, $m$ = 30 mg (d) $L_{p}$ = 4 mm, $m$ = 20 mg and (e) $L_{p}$ = 3 mm $m$ = 7 mg. (f) Simulated step responses comparing a system with only viscous damping (dashed, orange) and a system with additional quadratic damping (green). Viscous damping alone does not adequately reproduce the dynamic behavior of system, whereas quadratic damping approximates the step response more closely. |

| 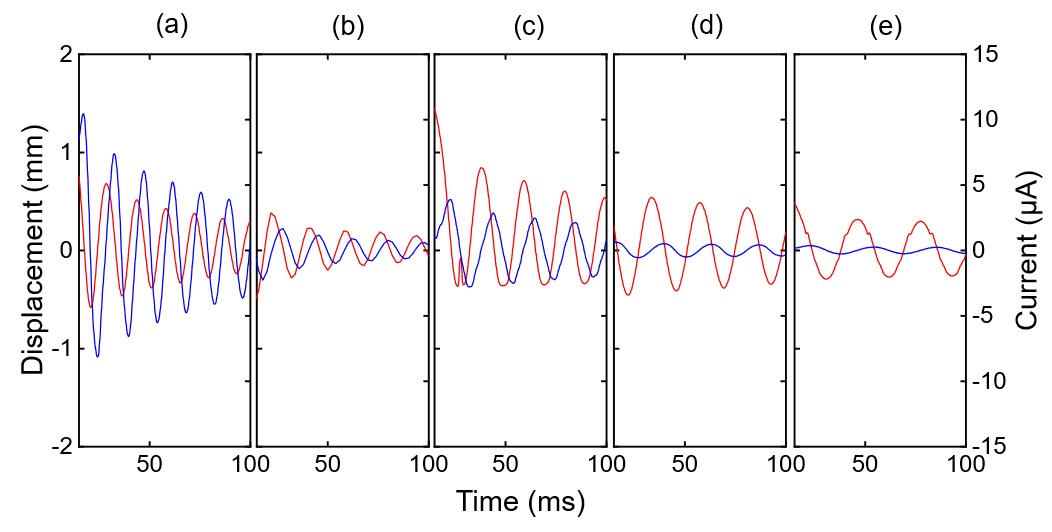 |
| --- |
| **Figure S2:** Experimental step responses of displacement (red) and current (blue) of cantilever devices for different length of the piezoelectric layer $L_{p}$ of (a) $L_{p}$ = 7 mm, (b) $L_{p}$ = 6 mm, (c) $L_{p}$ = 5 mm, (d) $L_{p}$ = 4 mm and (e) $L_{p}$ = 3 mm. The tip mass is 20 mg in each experiment. The displacement amplitudes $X$ and currents $I$ are used to determine the force factors $c_{i}=I/(2\pi fX)$ as explained in the Methods section of the manuscript. The frequency $f$ and amplitudes ($X, I$) are determined by a fit function based on a harmonic oscillator model. |


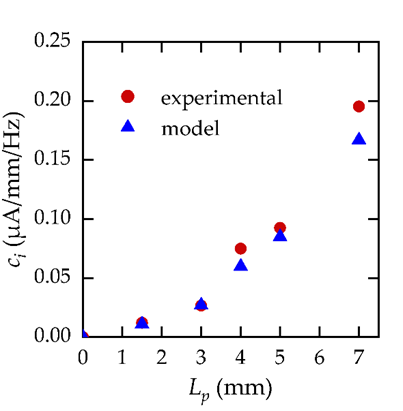


**Figure S3:** Force factors $c_{i}=I/(2\pi fX)$ determined from the experimental displacement amplitudes $X$ and currents $I$ shown in Fig. S2. The experimental force factors based on the piezoelectric properties at thickness 30 µm compare well with simulated results using datasheet values of corresponding bulk piezoelectric materials, which confirms that mechanical thinning did not affect the piezoelectric properties.

### A.3 Mechanical Power

| \| $\boldsymbol{L}_{\boldsymbol{p}}$ **(mm)**  (b) \| **Impact** \| **Structural** \| **EM** \| \| --- \| --- \| --- \| --- \| \| 3 \| 87.4%  (b) \| 12.4 % \| 0.2 % \| \| 4 \| 80.7 % \| 18.6 % \| 0.7 % \| \| 5 \| 42.6 % \| 54.8 % \| 2.6 % \| \| 6 \| 45.5 % \| 50.1 % \| 4.4 % \| \| 7 \| 4.6 % \| 83.4 % \| 12 % \|   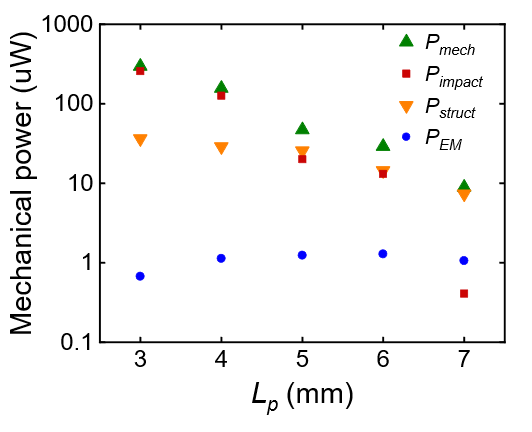  (a) |
| --- | --- | --- | --- | --- | --- | --- | --- | --- | --- | --- | --- | --- | --- | --- | --- | --- | --- | --- | --- | --- | --- | --- | --- | --- |
| **Figure S4:** Average mechanical power of the P-TM generators and the effect of the different damping mechanisms on power dissipation. (a) LEM simulation of average mechanical power in case of resonant self-actuation versus length of the piezoelectric layer $L_{p}$ at a source temperature of 100 °C. The mechanical power $P_{mech}$ is determined from the mechanical energy of the oscillator caused by the temperature-dependent change of magnetic attraction force $F_{mag}$. The mechanical power is dissipated through several mechanisms including impact of the cantilever tip with the magnet, structural damping and electromechanical (EM) damping (at the load resistance of 500 kΩ) giving rise to the power losses $P_{impact}$, $P_{struct}$ and $P_{EM}$, respectively. (b) Ratios of the different power losses: Impact - $P_{impact}/P_{mech}$, structural - $P_{struct}/P_{mech}$, and electromechanical - $P_{EM}/P_{mech}$. The strong increase of electromechanical damping with increasing $L_{p}$ from 0.2% to 12% is in line with the increase of the electromechanical coupling coefficient $k_{eff}^{2}$ (Table 1) showing a strong increase of power conversion. |

### A.4 Frequency Dependence of Capacitance

**Table S2:** Capacitance of the P-TM generators. The capacitance was measured using *BK Precision 880* LCR Meter at a voltage level of 0.6V and several frequencies. In the LEM and for the determination of $k_{eff}^{2}$, the values at 10 kHz are used.

| $\boldsymbol{L}_{\boldsymbol{p}}$ **(mm)** | $\boldsymbol{C}_{\boldsymbol{0}}$ **at 100 Hz** | $\boldsymbol{C}_{\boldsymbol{0}}$ **at 1 kHz** | $\boldsymbol{C}_{\boldsymbol{0}}$ **at 10 kHz** | $\boldsymbol{C}_{\boldsymbol{0}}$ **at 100 kHz** |
| --- | --- | --- | --- | --- |
| 7 | 13.95 nF | 13.47 nF | 12.93 nF | 12.00 nF |
| 6 | 11.51 nF | 10.95 nF | 10.50 nF | 9.71 nF |
| 5 | 10.11 nF | 9.72 nF | 9.36 nF | 8.67 nF |
| 4 | 9.14 nF | 8.61 nF | 8.17 nF | 7.61 nF |
| 3 | 7.88 nF | 7.62 nF | 7.12 nF | 6.67 nF |

### A.5 AC to DC Power Conversion

| 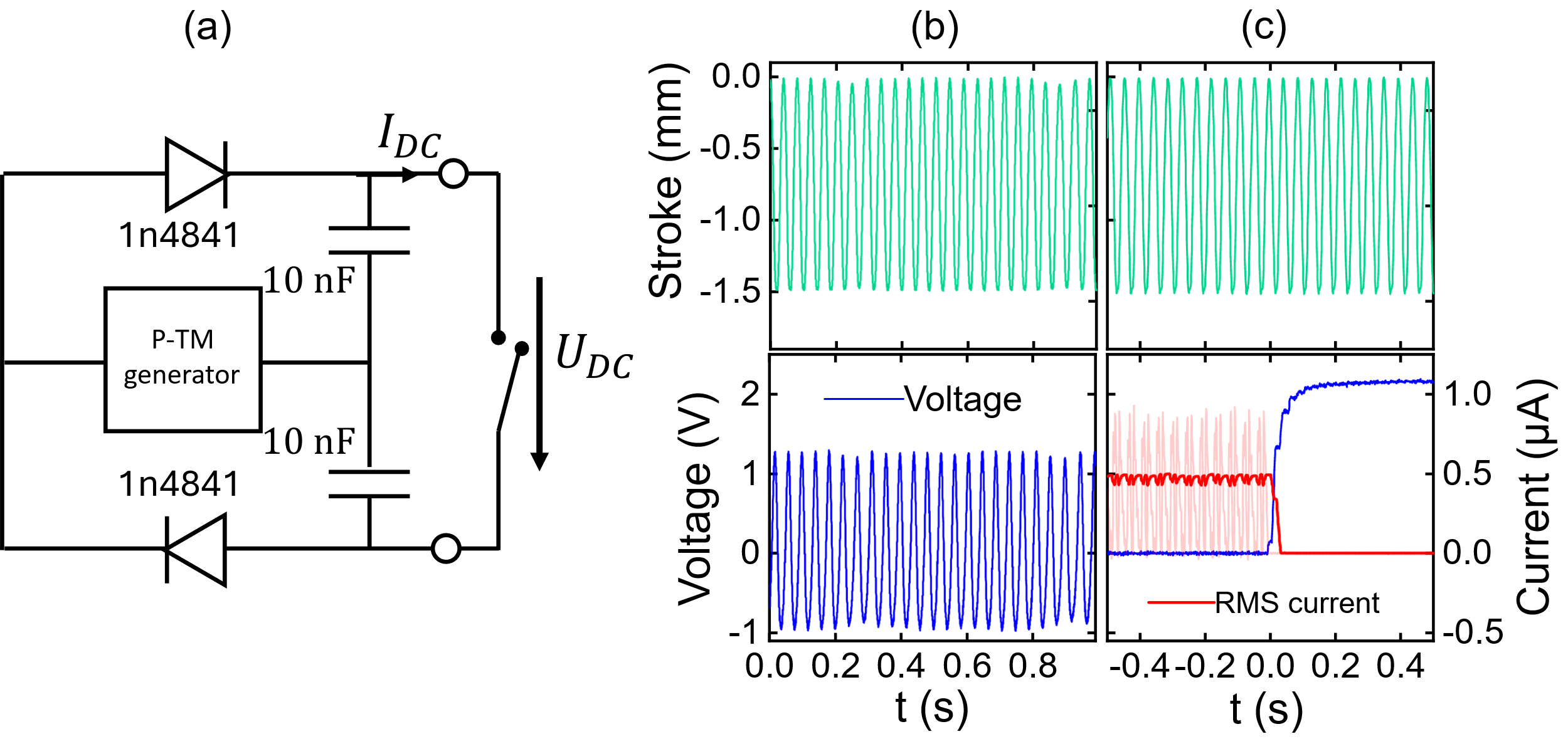 |
| --- |
| **Figure S5:** Experimental demonstration of AC to DC power conversion for a P-TM generator with a tip mass of 30 mg and a length of the piezoelectric layer $L_{p}$ = 5 mm at a source temperature of 105°C. (a) The rectification circuit utilizes two diodes and two capacitors to charge two 10 nF capacitors. A switch enables short-circuiting the output. (b) Without the circuit, the mechanical oscillation of the P-TM generator is converted to AC voltages in the range of -0.95 to 1.3 V. (c) After rectification, the open-circuit voltage $U_{DC}$ reaches 2.1V (t > 0). The generator yields a rectified RMS short-circuit current $I_{DC}$ of 0.47 µA (t < 0). This corresponds to an optimal DC power output $P_{opt}={U_{DC}I_{DC}}/4$ of 0.25 µW. With a device footprint of 0.2 cm², this corresponds to a DC power per footprint of 1.25 µW/cm². |

### A.6 Comparison of time-resolved experimental and simulated performances


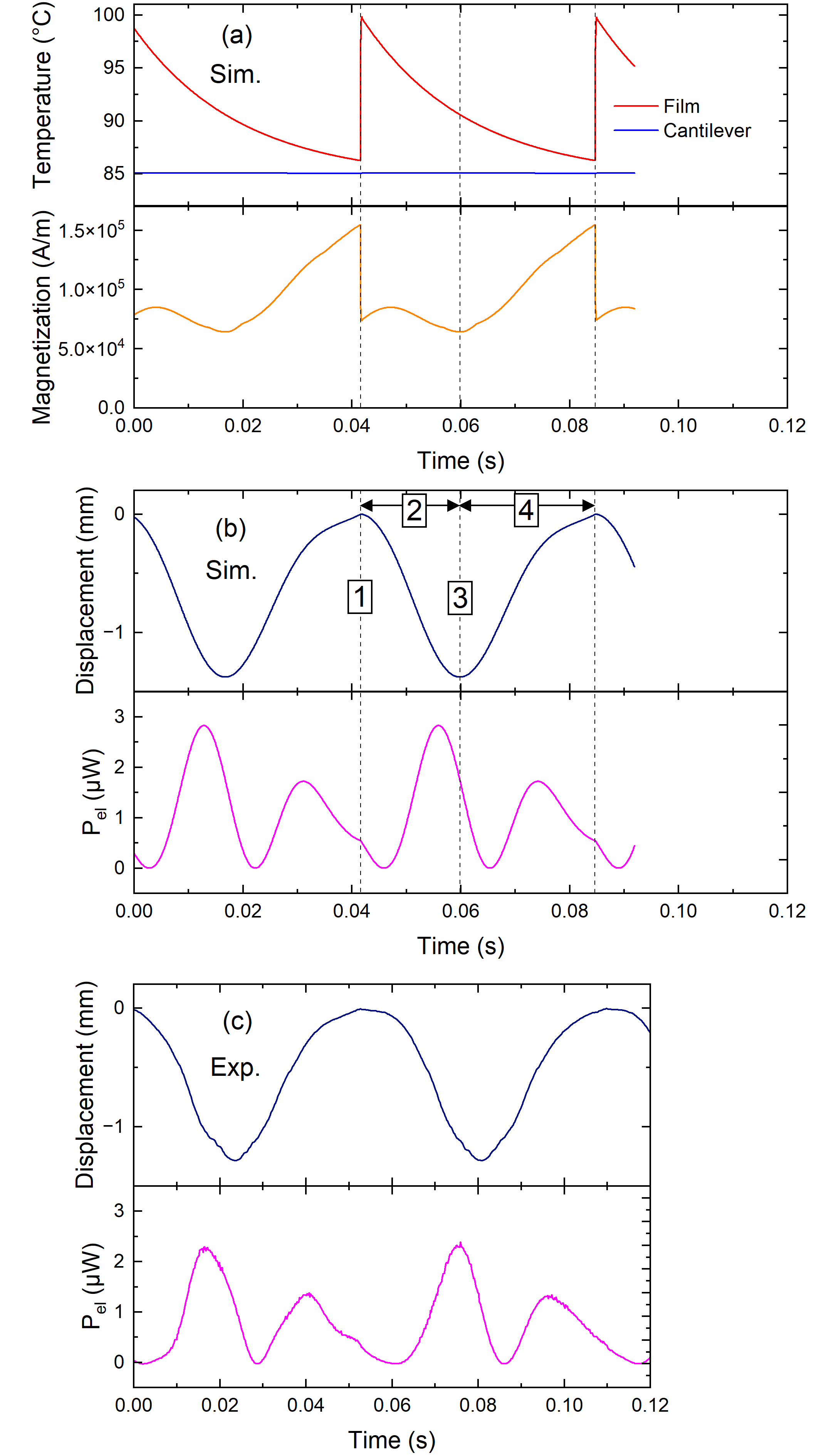


**Figure S6:** Time-resolved simulated and experimental signals of a P-TM generator with length of the piezoelectric layer $L_{p}$ of 7 mm. (a) Simulated temperatures of the TM film (*T_film_*) and cantilever (*T_cant_*)*,* as well as magnetization of the TM film, (b) simulated cantilever tip displacement and instantaneous electrical power $P_{el}$generated by electromechanical conversion through the P layer, (c) experimental cantilever tip displacement and instantaneous electrical power $P_{el}$. Four stages of cantilever motion 1-4 can be distinguished as highlighted: (1) Instant of heat transfer, when the Ni-Mn-Ga film is in mechanical contact to the heat source. In this case, the temperature rises sharply, which correlates with a sharp drop of magnetization. (2) Upon deflection from the magnet, the cantilever and P layer bend downwards to maximum strain corresponding to a maximum induced electrical power signal. The anomaly of magnetization is due to the interplay of temperature decrease and magnetic field decrease while moving away from the magnet. (3) Instant of maximum distance to the magnet, while the magnetization reaches a minimum. (4) Upon deflection toward the magnet, a second smaller maximum of induced electrical power signal occurs, when the cantilever and P layer bend upwards to maximum strain.

### A.7 Magnetic force measurement

| 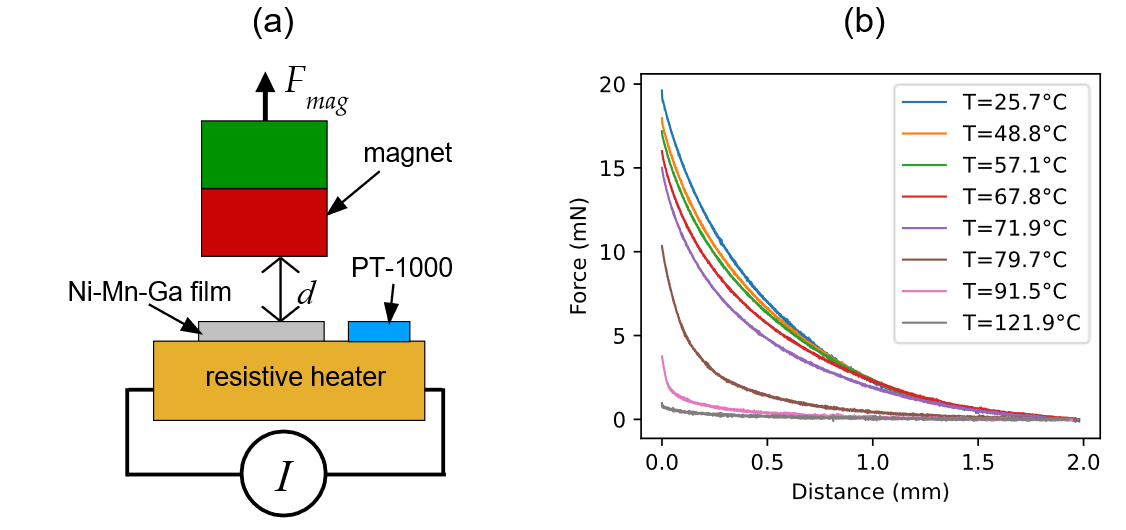 |
| --- |
| **Figure S7:** The lumped element model refers to a lookup table of measured magnetic forces as function of film temperature and distance between magnet and Ni-Mn-Ga film. Force measurements are conducted on a tensile testing machine using the *ZwickRoell Xforce HP 5N* force sensor. (a) Schematic of the setup showing the sample (a Ni-Mn-Ga film of 2 mm x 2 mm x 10 µm size) and the magnet. The TM film is heated using a resistive heater and a current source, while the temperature is measured by a PT-1000 sensor placed closely to the film. The heater remains stationary, while the magnet is moved at a speed of 0.5 mm/s. Quasi-stationary temperature conditions are obtained by increasing the temperature step wise and waiting until temperature change is negligible. At constant temperature, two measurement runs are conducted and the average of both is computed. A reference measurement without the Ni-Mn-Ga film is subtracted from all other measurements to eliminate parasitic magnetic forces. (b) Resulting force versus distance values at different temperatures, which are used in a lookup table for the lumped element model. |

### A.8 TM Cycle

*
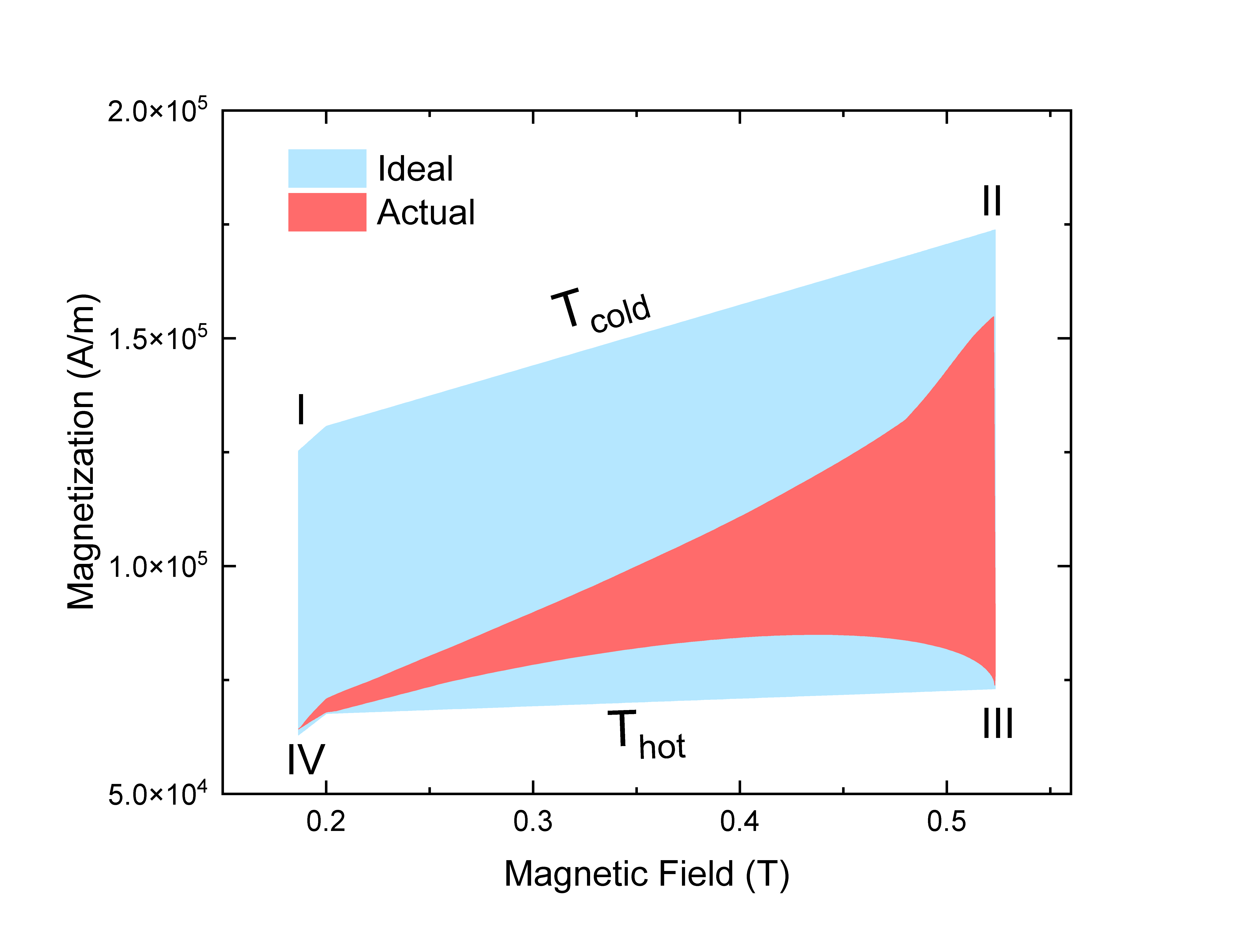
*

**Figure S8:** LEM simulation of the actual and ideal thermo-magnetic cycles of a P-TM generator with length of the P layer of 7 mm for a heat source temperature of 100 °C. In ideal case, the cycles consist of four steps I..IV, whereby II→III and IV→I denote heating and cooling steps at fixed magnetic fields corresponding to the ferromagnetic transition and I→II and III→IV assume that the change of magnetization for increasing and decreasing magnetic field occurs isothermally. The simulated cycle of the TM generator deviates from the idealized case due to non-isothermal magnetization change during oscillatory motion. The enclosed areas are used to determine the magnetic energy $E_{mag}$.

### A.9 Convection Coefficient

| 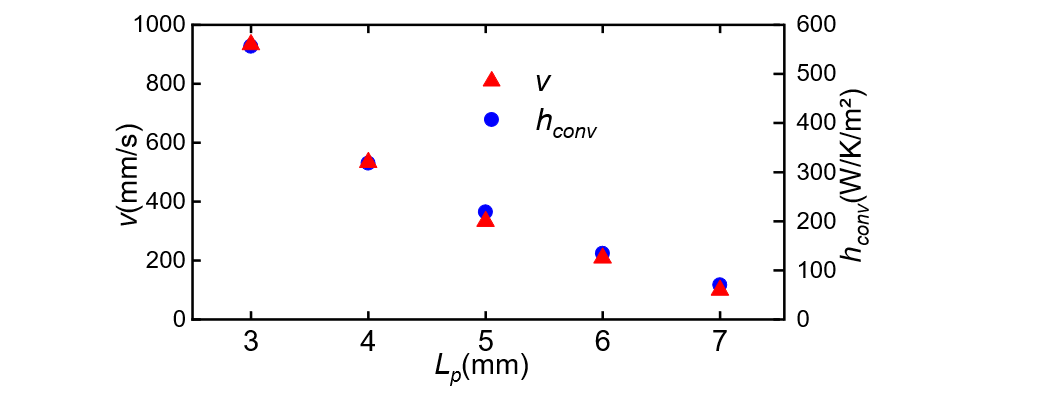 |
| --- |
| **Figure S9:** Oscillation speed $v$ and effective convective heat‑transfer coefficient $h_{conv}$ for P-TM generators with different lengths of piezoelectric layer $L_{p}$ at a source temperature *T_source_* of 100 °C. The experimental displacement amplitude $X_{op}$ (which accounts for half of the stroke) and oscillation frequency $f_{op}$ are used to determine the oscillation speed $v=2\pi f_{op}X_{op}$. For increasing length $L_{p}$, the oscillation speed $v$ decreases due to the decreasing stroke and frequency. The effective convective heat‑transfer coefficient $h_{conv}$ correlates with the oscillations speed $v$. |
